# Affective touch and face recognition: effects on memory and metacognitive performance

**DOI:** 10.1038/s41598-026-43969-9

**Published:** 2026-03-31

**Authors:** Madeleine Bregulla, Julian Packheiser, Christian J. Merz, Gerald Echterhoff, Dirk Scheele

**Affiliations:** 1 Department of Social Neuroscience, Center of Medical Psychology and Translational Neuroscience, Faculty of Medicine, Ruhr University Bochum, Bochum, Germany; 2 Research Center One Health Ruhr of the University Alliance Ruhr, Ruhr University Bochum, Bochum, Germany; 3 Department of Cognitive Psychology, Institute of Cognitive Neuroscience, Faculty of Psychology, Ruhr University Bochum, Bochum, Germany; 4 Social Psychology, Faculty of Psychology, University of Münster, Munster, Germany; 5 Faculty of Psychology, New School for Social Research, New York, NY USA

**Keywords:** Affective touch, Face memory, Metacognitive sensitivity, Social evaluations, Neuroscience, Psychology, Psychology

## Abstract

Episodic memories can be shaped by various contextual factors. While social and sensory cues such as odors and music have been shown to influence encoding and retrieval, the role of tactile information remains unclear. In this preregistered study, we investigated the effects of affective touch on face memory. 57 healthy adults (40 women) completed the Cambridge Face Memory Tests and the Social Touch Questionnaire to assess general face recognition ability and attitudes toward social touch. During encoding, participants viewed neutral faces while receiving either static, dynamic, or no touch from a hidden experimenter and rated each face’s attractiveness and trustworthiness. Recognition was tested two days later. Outcome measures included recognition accuracy, metacognitive sensitivity (i.e., the ability of confidence ratings to distinguish between correct and incorrect responses), and judgments of attractiveness and trustworthiness. No significant differences emerged between touch conditions, and neither face recognition ability nor attitudes toward touch moderated these effects. Bayesian analyses provided moderate to strong evidence for the absence of touch effects on recognition accuracy and confidence ratings, whereas evidence regarding metacognitive sensitivity and trustworthiness evaluations was inconclusive. Overall, the findings suggest that brief social touch in a controlled laboratory context has limited measurable effects on face memory, and that richer social context may be required for touch to influence memory processes.

**Supplementary Information:**

The online version contains supplementary material available at 10.1038/s41598-026-43969-9.

## Introduction

Human beings are tactile by nature. The sense of touch is one of the first senses to develop and is how children first explore their environments^1^. Everyday social interactions are replete with tactile gestures, from handshakes to hugs^2^. Touch plays a crucial role in attachment, fostering and strengthening social bonds^3,4^. It can positively influence sleep^5^, function as a stress buffer, and provide anxiolytic or comforting effects^6–8^. In fact, metanalytical evidence indicates that touch interventions have positive effects on both mental and physical health^9^. Additionally, social touch can be used as a communicative tool to convey (emotional) messages, especially in close relationships^10,11^.

On a physiological level, discriminative aspects of touch like pressure and temperature are mediated by myelinated Aβ-fibers, while the affective and hedonic qualities of touch are primarily encoded by unmyelinated C-tactile fibers that can be found in glabrous skin^12,13^. These fibers are especially reactive to slow, caress-like touches at speeds of between 1 and 10 cm/s^14^. This CT-optimal touch is perceived as pleasant^15^ and is intuitively used by people to caress loved ones^16^.

Numerous studies have shown that casual touches can alter people’s behavior and judgements, including a famous study where waitresses received higher tips after touching patrons^17^ and another where library patrons had more favorable opinions of said library after being touched by the librarian^18^. This phenomenon is referred to as “Midas-Touch” and describes the increase in positive affect and prosocial behavior after touch^19^. Beyond these pioneering field studies, this effect has been demonstrated in various contexts, including increased prosocial behavior in a dictator game following experimenter-administered hand touch^20^, reduced negative perception of images when participants received touch^21^, and heightened approachability ratings of faces after CT-optimal touch compared to fast, not-CT optimal or no touch^22^. In previous studies, priming with images of vicarious social touch enhanced attention to faces and facial expressions in subsequently presented scenes^23^ and increased neural responses to emotional faces^24^. Based on these findings, the authors proposed that touch may also facilitate subsequent memory for those faces. Additionally, CT-optimal touch has a social sharpening effect on presented faces, amplifying the perceived friendliness of smiling faces while diminishing these qualities in frowning faces^25^. Thus, social touch, especially CT-optimal touch, appears to not only intensify the emotionality of stimuli but also enhance their social significance. However, while previous research has examined the effects of touch on various aspects of social interactions and individual well-being, its impact on (social) memory has so far been neglected.

Different sensory modalities have been linked to enhanced memory performance. So-called Proustian phenomena are autobiographical memories that are triggered by olfactory stimuli^26^, and emotionally touching music can enhance face memory performance^27^. In paradigms using multisensory contexts to test memory performance, items presented together with task irrelevant information in a different sensory modality were remembered better^28^. Stronger connectivity between face-processing networks with other networks involved in visual and auditory processing constitutes also a predictor for better face memory performance^29^. Furthermore, social contexts can also modulate memories. Verbal memory performance is enhanced for words presented as feedback from another person compared to words presented without a social context^30^. Additionally, recognition memory improves not only for self-relevant items, but also for words relevant to a confederate present during encoding^31^. Social contexts during encoding can even lead to the generation of false memories for partner-relevant stimuli^32^.

Based on findings that sensory stimuli can enhance memory performance and social contexts can modulate encoding, we designed a study to investigate the effects of social touch during encoding on recognition accuracy and metacognitive sensitivity (i.e. how efficiently confidence judgements distinguish between correct and incorrect judgements) in a face rating paradigm with a surprise recognition test two days later. Since especially CT-optimal touch is associated with feelings of pleasantness^15^ and neural activations of reward-related brain areas^33^, the positive affect created by the tactile stimulation could have a positive effect on memory^34,35^ that goes beyond the effects of additional sensory stimulation. In line with the “Midas-touch” and social sharpening effects, we explored touch effects on the affective evaluation of the faces (i.e. trustworthiness and attractiveness ratings) as a potential mechanism for memory effects. We used neutral faces as stimuli because prior research has shown no interaction between touch effects and facial emotionality at the neural level^24^ and because CT-optimal touch has been found to influence evaluations of neutral faces^22^. To disentangle effects specific to CT-optimal stimulation from more general multisensory influences, we contrasted dynamic, CT-optimal touch with static touch, thereby isolating the contribution of affective, velocity-dependent tactile input beyond the mere addition of concurrent tactile stimulation.

We hypothesized that social touch during encoding would positively affect participants’ ability to correctly remember unfamiliar, neutral faces (Hypothesis 1), and that social touch would improve the feeling of confidence in those memories (Hypothesis 2). Furthermore, we expected that social touch would increase metacognitive sensitivity (Hypothesis 3). Given that touch may evoke varying emotional responses based on an individual’s attitude towards being touched, we hypothesized that touch effects would be moderated by personal attitudes towards social touch (Hypothesis 4). Moreover, we expected these effects to be stronger for dynamic touch compared to static touch (Hypothesis 5). Furthermore, we explored how touch affects the perceived attractiveness and trustworthiness ratings of faces and whether this effect is moderated by participants’ attitudes towards social touch or baseline attractiveness and trustworthiness ratings.

## Materials and methods

### Participants

We recruited healthy individuals aged between 18 and 65 years who had no neurological or mental disorders, no excessive scar tissue on their forearms or other conditions affecting sensory perception, normal or corrected-to-normal vision, and proficient German language skills to ensure comprehension of study materials. Additionally, we restricted participation to individuals who were not currently in a romantic relationship to minimize potential socially desirable responding or influences of relationship status and commitment on attractiveness ratings^36^. Prior research has shown that relationship status can affect preferred interpersonal distance^37^, accordingly, we could not rule out the possibility that it might also influence perceptions of touch administered by unfamiliar strangers.. The final sample consisted of 57 participants (17 males) with an average age of 27.13 years (SD = 8.16). 54 participants identified as heterosexual. Participants all gave informed consent. The study was approved by a local ethics board at Ruhr-University Bochum (ID 863) and was conducted in accordance with the declaration of Helsinki.

We conducted an a-priori power analysis for mixed models with two levels through a simulation approach with the R software package SIMR^38^. Based on the approach outlined in Arend and Schäfer^39^, we simulated different second level sample sizes (40, 50, 60, 70, 80) for the number of participants while keeping the first level sample size constant for the three different touch conditions. For a significance-level of α = 0.05, a medium effect size, at a second level sample size of N = 50, we would reach 81% (95% confidence interval 78.43, 83.39) power for a medium ICC and 79.30% (95% confidence interval: 76.65, 81.77) at a large ICC. We would reach 85.60% (95% confidence interval 83.24, 87.72) with a medium ICC and 85.20% (95% confidence interval 82.85, 87.34) power with a large ICC at a second-level sample size of N = 60. We thus planned to test 60 participants. Due to violations of study inclusion criteria, three participants had to be excluded after termination of data collection, resulting in a final sample size of 57 participants, which still left us with a satisfactory power level of above 80%.

### Experimental procedure

Participants took part in two experimental sessions separated by approximately 48 h. Both appointments took place in the same test room. During the first session, they first completed both the female and male versions of the Cambridge Face Memory Tests Long Version (CFMT + ^40^ and fCFMT + ^41^). Afterwards, they were instructed to view and rate the trustworthiness and attractiveness of 96 neutral face images (half of them female) from the Oslo face database^42^ presented on a computer screen. Trustworthiness and attractiveness ratings for the presented faces were given on a scale from 1 (“Not trustworthy/attractive”) to 10 (“Very trustworthy/attractive”). The first presentation block of all faces occurred without touch (t1). During the second (t2) and third presentation block (t3), each face was paired with one of three touch conditions (no touch, dynamic touch, or static touch) and participants were instructed to imagine that the touch was performed by the person on screen. The faces were presented for 4 s each, followed by the ratings and a 2-s-long fixation cross. Faces were presented in a randomized order, and the pairing of a face with a touch condition was randomized across participants but remained constant within each individual participant across blocks. For all three touch conditions, participants placed their arm behind a curtain so they could be touched without seeing the movement of the hand of the experimenter. In the dynamic touch condition, a hidden experimenter stroked the participant’s upper forearm for 4 s at a slow speed of approximately 5cm/s, targeting CT-fibers in the skin. To maintain consistent stroking speeds, the experimenter followed audio cues while stroking a marked 15 cm area on the participant’s forearm. During the static touch condition, the experimenter placed their hand gently on the participant’s forearm in the middle of the marked area for the duration of the stimulus presentation. For all touches, the experimenter wore a satin glove to ensure touch consistency across participants. The hand was lifted at the end of an audio cue. Based on evidence that touch administered by a presumably female experimenter is generally perceived as more pleasant^43^ and more acceptable irrespective of the recipient’s gender^44,45^, as well as the fact that participants could not see the experimenter behind the curtain, all touch was delivered by the same female research assistant. To facilitate the imagination of being touched by the face presented on screen, this assistant was not the experimenter who conducted the remainder of the session, thereby reducing potential interference with the imagined social source of the touch. At the end of t2 and t3, participants rated the perceived pleasantness of the touch on a scale from 1 (“not pleasant”) to 5 (“very pleasant”).

**Fig. 1:**
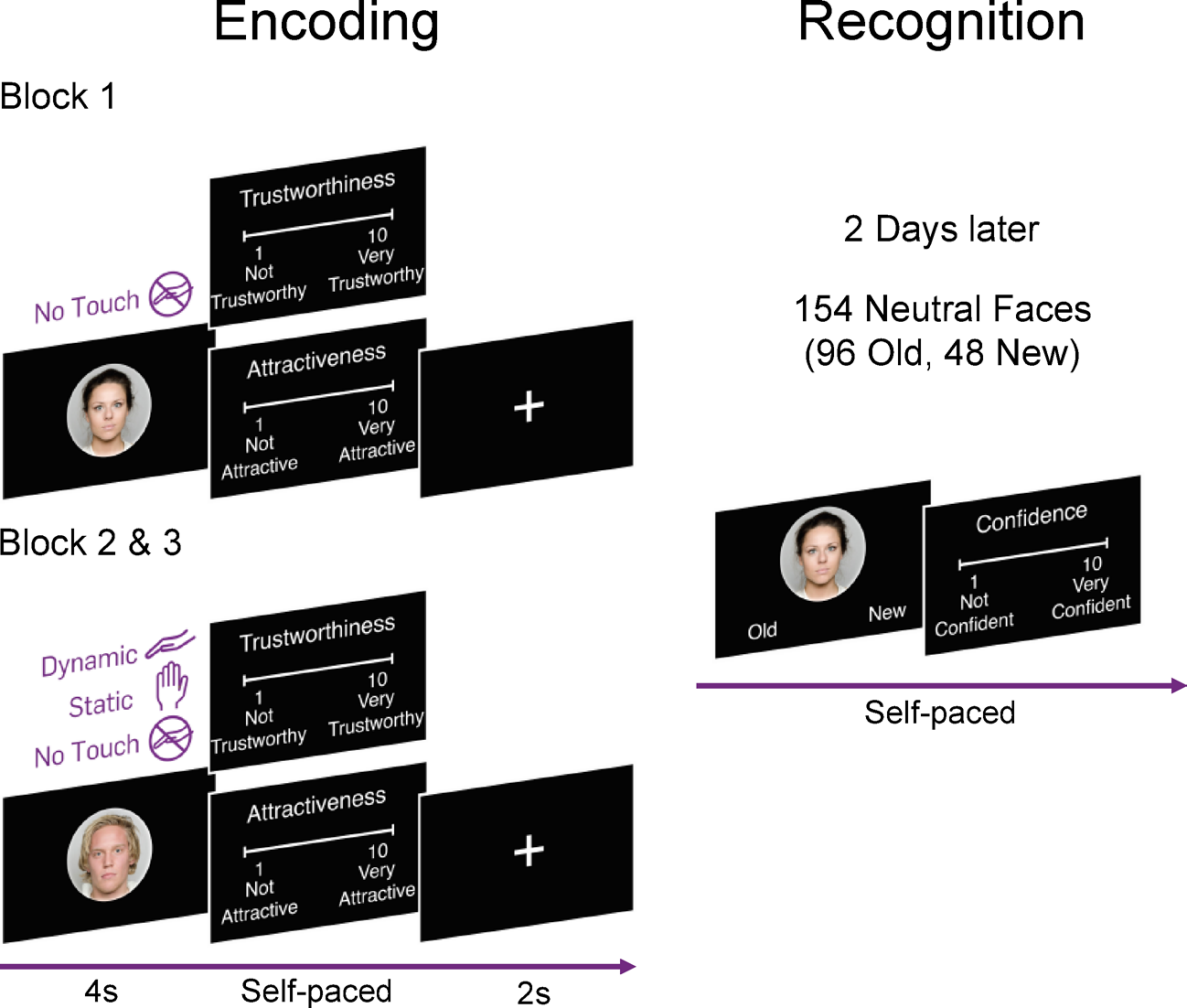
Experimental procedure of the face recognition paradigm. The faces originated from the Oslo Face Database^42^.

In the second session, held 48 h later, participants underwent a surprise recognition test. They were presented with the 96 previously seen faces and 48 new faces (24 female) in a randomized order, and asked to indicate whether they had seen each face before, as well as to rate their confidence in their judgements. There was no time limitation for trustworthiness and attractiveness ratings during encoding, the decision whether they saw an old or new image, or the confidence ratings (self-paced). An overview of the task is shown in Fig. 1, with further details about the experimental protocol available in the online supplementary information (SI). We thus employed a within-subjects design in which touch condition served as the primary factor, comprising of two (touch/no touch) or three levels (no touch/static touch/dynamic touch) depending on the tested hypothesis.

Recognition accuracy was measured with d prime (d’), a standardized measure of discriminability unaffected by response biases^46^. After indicating whether they had seen a face during the previous laboratory session, participants rated their confidence in their judgement on a scale from 1 (“Not confident”) to 10 (“Very confident”). Metacognitive efficiency was measured with meta d’, a standardized measure that quantifies the efficacy of confidence ratings in discriminating between correct and incorrect judgements^47^. Meta d’ was computed using the approach outlined in Maniscalco and Lau^47^ and the MATLAB code published by the authors.

We measured participant’s attitude towards social touch with the Social Touch Questionnaire (STQ^48^), self-reported memory performance and difficulties with the Multifactorial Memory Questionnaire (MMQ^49^), sensory imagination skills with the Plymouth Sensory Imagery Questionnaire (PSI-Q^50^), and face memory skill through performance on the Cambridge Face Memory Test Long Version (CFMT + ^40^ and fCFMT + ^41^). Participants completed the questionnaires on both experimental sessions. To prevent anticipation of the recognition test, memory-related questionnaires were administered only after participants had finished the recognition task. Questionnaires that were given to participants but not considered for this study are listed in the SI.

### Statistical analysis

The analysis plan was preregistered before conducting any analysis (10.17605/OSF.IO/F8T3K). To account for the multilevel data structure – with face stimuli nested within touch conditions which in turn were nested within participants – we employed linear mixed models to analyze the data using the statistical software R within the RStudio environment^51,52^. Additionally, we complemented frequentist analyses with Bayesian analyses to evaluate evidence for the null hypotheses.

Touch condition (touch and no touch) was modeled as a fixed effect to predict d’ (Hypothesis 1), confidence ratings (Hypothesis 2), and meta-d’ (Hypothesis 3). For hypothesis 4, we added the interaction term touch condition * STQ-score to the models that were evaluated for hypotheses 1 to 3 as a fixed effect. For hypothesis 5, the factor touch condition in the previous models was subdivided into three levels (dynamic, static and no touch). For each of the models, fixed effects of grand-mean centered MMQ, PSI-Q and (f)CFMT + scores, sex of the presented face, age and sex of the participant were considered as covariates.

The models were fitted using restricted maximum-likelihood and with participants as random effect factors, allowing for intercepts to vary across participants. To identify the maximal random effects structure supported by the data, we made use of a stepwise approach^53^.

For the exploratory analyses, we used the rating difference between baseline and the mean of the two timepoints with the different touch conditions (i.e. mean(t2,t3)  – t1) for attractiveness and trustworthiness respectively as dependent variable in the model and added the touch condition and the interaction term touch condition * STQ-score as fixed effects. Additionally, to analyze the moderating effect of the baseline ratings for each face, the interaction term touch condition * baseline rating of attractiveness or trustworthiness of each face was added as a fixed effect.

We used the standard *p* < 0.05 criterion for determining statistical significance. Additionally, we computed Bayes factors for the analysis, testing a scaling factor from small to large effects (e.g. d = 0.3, 0.5 and 0.7). We considered *BF*_*01*_ > 3 as moderate evidence, *BF*_*01*_ > 10 as strong evidence, and *BF*_*01*_ values between 1/3 and < 3 as insufficient evidence to draw a conclusion on the null hypothesis^54^.

For further details of the statistical analyses, see the SI. In the study itself, we will report results of the Bayesian analyses with a prior that assumes a medium effect size as outlined in the power analysis. The model estimates and Bayes factors for the other tested priors are reported in the supplement.

## Results

### Memory effects

**Fig. 2:**
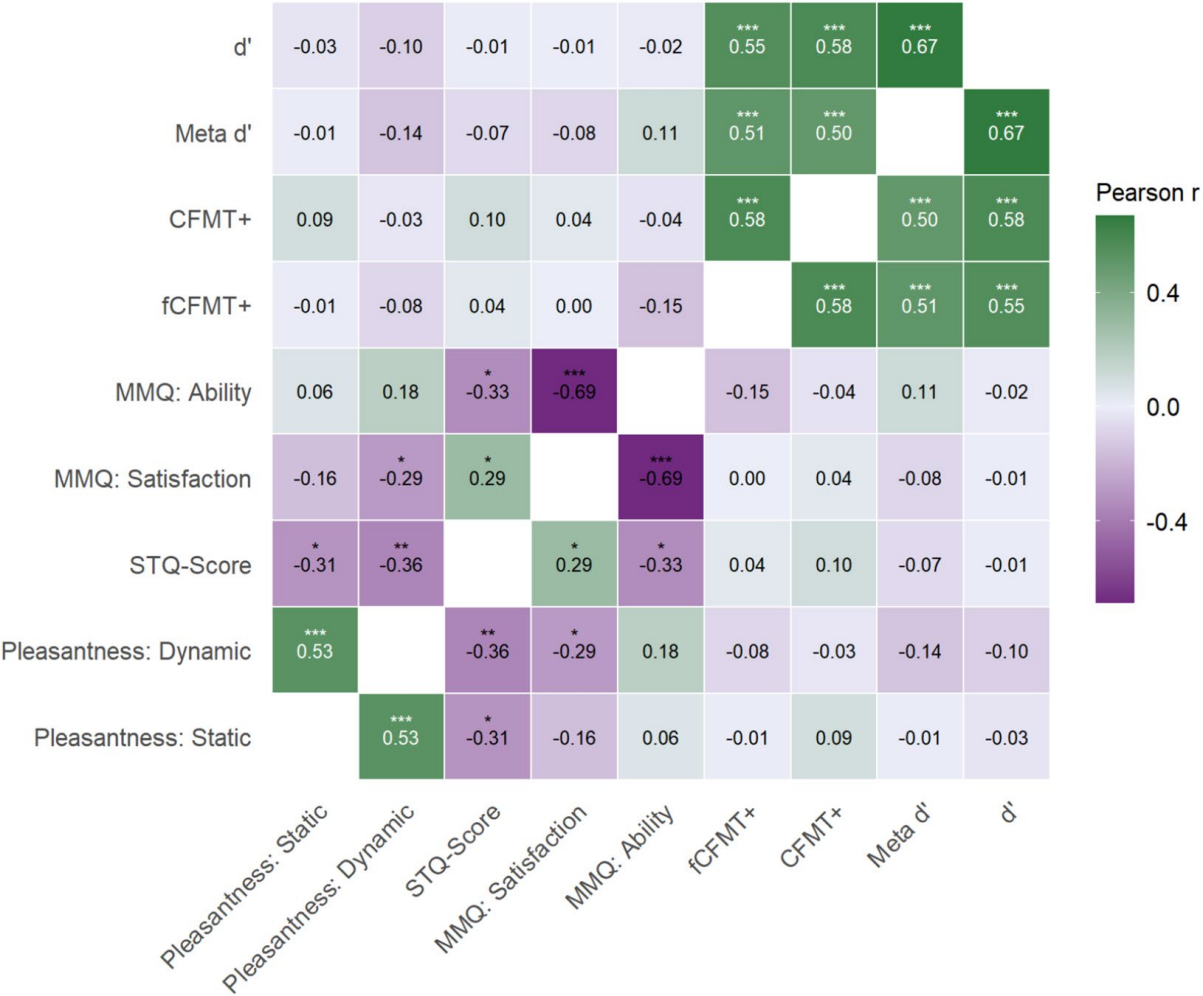
Correlational heatmap (memory and metacognitive sensitivity averaged across touch conditions). *STQ* Social Touch Questionnaire, *MMQ* Meta Memory Questionnaire, *PSI-Q* Plymouth Sensory Imagination Questionnaire, *CFMT* + Cambridge Face Memory Test Long Version, *fCFMT* + female Cambridge Face Memory Test Long Version. Higher scores of the STQ indicate higher touch aversion. *p < 0.05, **p < 0.01, ***p < 0.001.

Across the touch conditions, participants recognized 80.21% (*SD* = 14.02) of the previously shown faces and identified 94.96% (*SD* = 2.99) of the new distractor images. Participants perceived both touch conditions equally as relatively pleasant (static: *M* = 3.55, *SD* = 0.95; dynamic: *M* = 3.43, *SD* = 1.08; *t*(56) = – 0.87, *p* = 0.39, *BF*_*01*_ = 4.83). As expected, participants with better performance in both versions of the CFMT showed significantly better face recognition accuracy and metacognitive sensitivity (see Fig. 2). Likewise, higher touch aversion was associated with reduced pleasantness ratings for both dynamic (*r* = – 0.36, *p* = 0.01, *BF*_*01*_ = 0.16) and static touch (*r* = – 0.31, *p* = 0.02, *BF*_*01*_ = 0.42). Higher touch pleasantness ratings for static or dynamic touch were not significantly associated with better recognition accuracy (static: *r* = – 0.01, *p* = 0.97, *BF*_*01*_ = 6.05; dynamic: *r* = – 0.14, *p* = 0.29, *BF*_*01*_ = 3.54), confidence ratings (static: *r* = 0.00, *p* = 0.98, *BF*_*01*_ = 6.05, dynamic: *r* = – 0.08, *p* = 0.55, *BF*_*01*_ = 5.07), or metacognitive sensitivity (static: *r* = 0.01, *p* = 0.95; *BF*_*01*_ = 6.04; dynamic: *r* = – 0.23, *p* = 0.08, *BF*_*01*_ = 1.37).

**Fig. 3:**
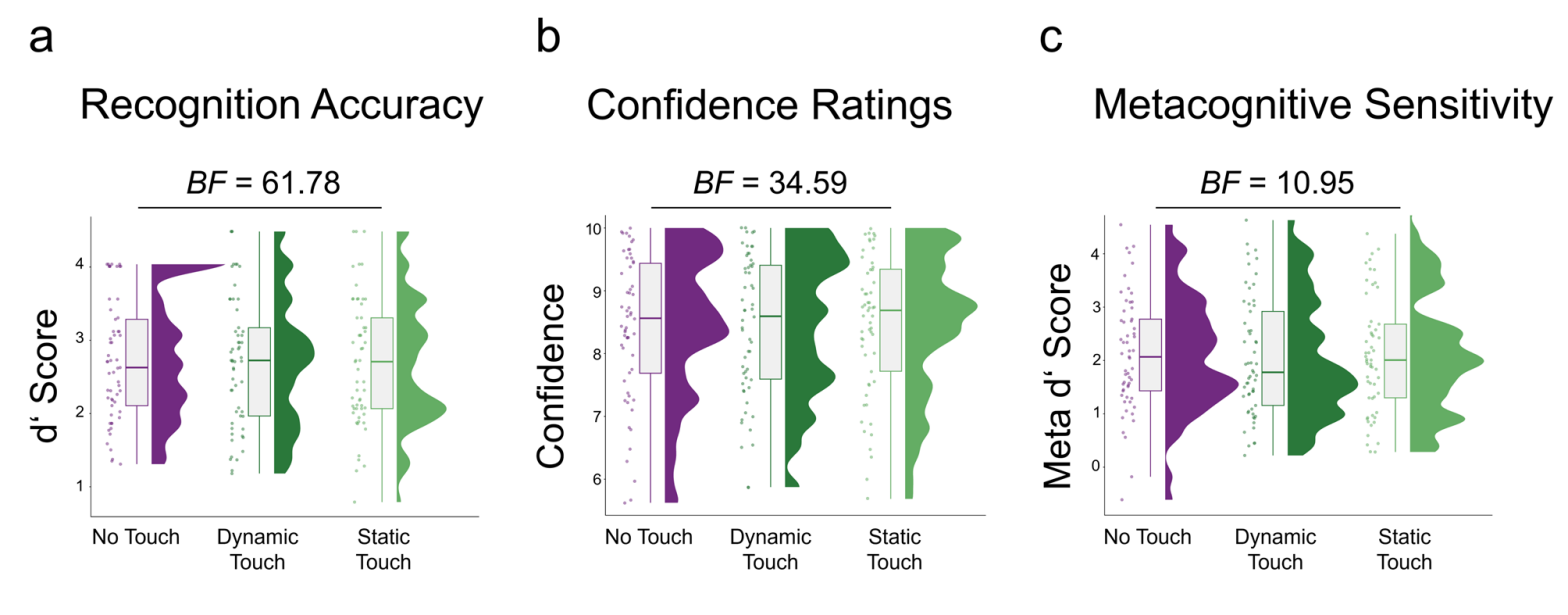
Bayesian analyses provided strong evidence that there was no significant effect of static or dynamic touch on recognition accuracy as measured with d’ (**a**), confidence ratings (**b**) or metacognitive sensitivity as measured with meta d’ (**c**). The boxes in the plots show the interquartile range (IQR) of the data, where the upper and lower edges correspond to the first and third quartiles respectively. The horizontal line inside the boxes represents the median, while the whiskers outside the boxes indicate the range of non-outlier data points (i.e. data not exceeding 1.5 times the IQR range).

Importantly, there was no significant effect of touch condition on recognition accuracy (*t*_(99)_ = 0.22, *p* = 0.82, β = 0.01, 95% CI [– 0.09, 0.12]), confidence ratings (*t*_(99)_ = 0.57, *p* = 0.57, β = 0.03, 95% CI [– 0.06, 0.12]), or metacognition (*t*_(99)_ =  – 0.52, *p* = 0.60, β = – 0.06, 95% CI [– 0.29, 0.17]). Bayesian analyses revealed moderate evidence for the null hypothesis both for recognition accuracy (*BF*_*01*_ = 8.33) and confidence ratings (*BF*_*01*_ = 6.79), for metacognitive sensitivity, the evidence can however only be considered inconclusive (*BF*_*01*_ = 2.63). Exploratory analyses for dynamic and static touch confirmed this null effect (see Fig. 3). Estimates for the dynamic (*t*_(153)_ = 0.13, *p* = 0.90, β = 0.01, 95% CI [– 0.12, 0.13]) and static touch (*t*_(153)_ = – 0.20, *p* = 0.84, β = – 0.01, 95% CI [-0.14, 0.11]) compared to no touch revealed no significant differences between touch conditions for recognition accuracy. We observed strong evidence for the null hypothesis (*BF*_*01*_ = 61.78). Confidence ratings were once again not predicted by the factor touch. Estimates for dynamic (*t*_(153)_ = 0.34, *p* = 0.73, β = 0.02, 95% CI [– 0.10, 0.13]) and static touch (*t*_(153)_ = 0.54, *p* = 0.59, β = 0.03, 95% CI [– 0.08, 0.15]) compared to no touch showed no significant differences between touch conditions. The evidence for the null hypothesis can be considered strong (*BF*_*01*_ = 34.59). Likewise, metacognitive estimates for dynamic (*t*_(99)_ = 0.06, *p* = 0.95, β = 0.01, 95% CI [– 0.20, 0.21]) and static touch (*t*_(153)_ = – 0.57, *p* = 0.57, β = – 0.06, 95% CI [– 0.26, 0.15]) compared to no touch showed no significant differences between touch conditions. The model yielded strong evidence for the null hypothesis (*BF*_*01*_ = 10.95).

### Moderation by attitudes towards touch

Attitudes towards social touch had neither a significant main effect nor a significant moderation effect on recognition accuracy (STQ: *t*_(97)_ = – 0.67, *p* = 0.51, β = – 0.08, 95% CI [– 0.31, 0.15]; condition*STQ: *t*_(97)_ = 0.38, *p* = 0.81, β = 0.01, 95% CI [– 0.09, 0.12]), confidence ratings (STQ: *t*_(97)_ = – 0.67, *p* = 0.51, β = – 0.08, 95% CI [– 0.35, 0.18]; condition*STQ: *t*_(97)_ = 0.06, *p* = 0.95, β = 0.003, 95% CI [– 0.09, 0.09]), or metacognitive sensitivity (STQ: *t*_(97)_ =  – 0.002, *p* = 0.91, β = – 0.002, 95% CI [– 0.25, 0.25]; condition*STQ: *t*_(97)_ = – 1.23, *p* = 0.22, β = – 0.14, 95% CI [– 0.37, 0.09]). Evidence for the null hypothesis was strong for all three models (*BF*_*01*_ > 10). Separate analyses for static and dynamic touch did not reveal any significant main or interaction effect of the STQ (all *p*s > 0.05) and provided strong evidence for the null hypothesis (*BF*_*01*_ > 10).

### Social judgements

**Fig. 4:**
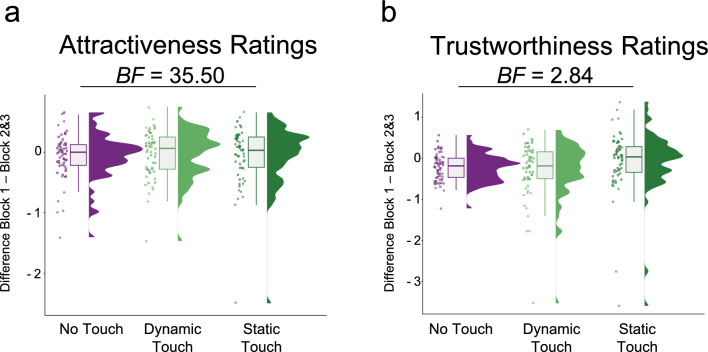
Bayesian analyses provided strong evidence that neither dynamic nor static touch had an effect on attractiveness ratings (**a**). Evidence for the absence of an effect on trustworthiness ratings is inconclusive (**b**). For both attractiveness and trustworthiness, the mean ratings for the images after touch (Block 2 & 3) were subtracted from the baseline rating without touch (Block 1). The boxes in the plots show the interquartile range (IQR) of the data, where the upper and lower edges correspond to the first and third quartiles respectively. The horizontal line inside the boxes represents the median, while the whiskers outside the boxes indicate the range of non-outlier data points (i.e. data not exceeding 1.5 times the IQR range).

We found no significant effect of touch condition on attractiveness ratings (*t*_(99_) = 0.17, *p* = 0.87, β = 0.03, 95% CI [– 0.31, 0.36]) or trustworthiness ratings (*t*_(99)_ = 0.18, *p* = 0.86, β = 0.03, 95% CI [– 0.33, 0.40]). Bayesian analysis revealed moderate evidence for the null hypothesis for attractiveness (*BF*_*01*_ = 5.85). However, evidence for the null hypothesis for trustworthiness was inconclusive (*BF*_*01*_ = 0.94). Similarly, separate analyses for dynamic and static touch showed no significant effect on attractiveness ratings (dynamic touch: *t*_(153)_ = 0.39, *p* = 0.70, β = 0.06, 95% CI [– 0.25, 0.37]; static touch: *t*_(153)_ = – 0.06, *p* = 0.95, β = – 0.01, 95% CI [– 0.32, 0.30]) or trustworthiness ratings (dynamic touch: *t*_(153)_ = – 0.82, *p* = 0.42, β = – 0.14, 95% CI [– 0.47, 0.19]; static touch: *t*_(153)_ = 1.13, *p* = 0.26, β = 0.19, 95% CI [-0.14, 0.52]; see Fig. 4). Evidence for the null hypothesis was strong (*BF*_*01*_ = 35.50) for attractiveness and inconclusive for trustworthiness (*BF*_*01*_ = 2.84).

Finally, neither the attitudes towards touch nor the baseline attractiveness or trustworthiness ratings significantly moderated the effects of touch (all *p*s > 0.05). There was strong evidence for the null hypotheses in all cases (*BF*_*01*_ > 10). The detailed results for the moderation analyses as well as complete estimates of fixed effects for all the models can be found in the SI.

## Discussion

In the present study, we investigated the effects of social touch on recognition accuracy, metacognitive sensitivity, as well as trustworthiness and attractiveness ratings in a face rating paradigm with a surprise recognition test two days later. Task performance was significantly correlated with general face recognition ability, while higher touch pleasantness ratings were significantly correlated with more favorable attitudes towards social touch. Contrary to our hypotheses, we did not find any significant influence of social touch – be it a slow caress or a static touch on the forearm – on any of our outcome measures. We also found no measurable influence of attitudes towards social touch on (meta)memory performance nor on the touch effects. Bayesian analyses provided moderate to strong evidence against the presence of at least medium-sized effects of touch on the examined outcomes. While brief touch may plausibly exert small effects in naturalistic contexts where interpersonal and situational nuances shape the interaction, in a highly standardized laboratory paradigm, we defined effects of at least moderate magnitude as the smallest effect size of practical relevance. In line with recent discussions on smallest effect sizes of interest in memory research, more tightly controlled and less ecologically valid paradigms typically warrant larger thresholds for what constitutes a theoretically or practically relevant effect^55^.

Our findings largely align with a recent study investigating the impact of touch on both memory performance and metacognitive sensitivity for emotional scenes^56^. In this study, participants viewed images while being either slowly stroked with a soft brush or sandpaper and performed a recognition task 20 min later. The authors did not find a significant effect of touch on recognition accuracy but reported lower levels of metacognitive sensitivity for trials with negatively valanced touch. Given that the touch administered in our study was perceived as pleasant, these results suggest that neither pleasant social nor non-social touch modulates short-term or long-term memory for faces or scenes.

Interestingly, in a previous study CT-optimal human touch increased the perceived friendliness of faces, but there was also no significant effect on attractiveness ratings^25^. Furthermore, CT-optimal touch administered by a rotary tactile stimulator only prevented a reduction in approachability observed for other faces that were not associated with touch over the course of the study^22^. Likewise, a short vibrating touch prime administered by a machine did not significantly alter affective responses to emotional faces measured with event related potentials and facial electromyogram^24^. Our moderation analyses revealed that the absence of a touch effect on attractiveness and trustworthiness ratings cannot be attributed to interindividual differences in the attitudes towards social touch. Collectively, these results point to a nuanced effect of touch on the evaluation of faces. Previously observed field experiment effects, wherein social touch leads to more positive evaluations of a person and a situation as well as increased prosocial behavior^19^ may not become evident in controlled laboratory experiments with brief touch. In fact, CT-optimal touch had no significant effect on prosocial behavior in economic laboratory tasks^57^, so the lack of influence of social touch on the social judgements in our study might similarly be impacted by the laboratory setting.

Furthermore, ample evidence indicates that the effects of social touch are highly context-dependent. For instance, hedonic evaluations and brain responses to social touch are significantly moderated by information about the presumed toucher^43,58^. The relationship with the toucher even modulates the effectiveness of touch as social support^59^. Thus, social touch may increase interpersonal trust only in certain contexts^60^. Context effects may therefore also contribute to the absence of touch effects on memory performance. In infants, affective touch performed by a parent, but not by an experimenter, was able to increased face discrimination^61^. In adults, slow caresses and prolonged static touch are both more commonly associated with familiarity rather than situations requiring the encoding of unfamiliar faces. It is therefore conceivable that the tactile stimulation did not influence encoding due to a mismatch between the touch context, which implied familiarity, and the task context, which implied meeting strangers. This mismatch may have led participants to process the tactile and visual elements separately, preventing the integration of information from both domains. Such integration could have facilitated a deeper level of processing and enhanced memory^28,29^.

Participants demonstrated high face recognition accuracy, raising the possibility that existing touch effects may have been obscured by ceiling effects. However, touch effects were not significantly moderated by memory performance, as independently assessed by the CFMT. To further explore this, future studies could investigate touch effects in samples with mild cognitive impairment or dementia. Additionally, we found no interaction between attitudes towards social touch and touch conditions. Nevertheless, given that previous traumatic experiences are associated with strong negative attitudes towards touch^62,63^, effects may differ in individuals with more severe traumatic experiences. Besides the role of differences in attitudes towards social touch as moderating factor, the moderating effects of interindividual differences such as hormonal effects through sex hormones, intake of oral contraceptives, or differences in oxytocin levels, as well as personality traits like extraversion should be investigated in future studies. Moreover, re-administering touch during retrieval could serve as a contextual cue and thereby influence memory performance and/or metacognitive sensitivity, given that memory is often enhanced when encoding and retrieval contexts are congruent^64^. To strengthen the associative link between touch and the face stimulus – and to more clearly isolate encoding-related effects from potential re-exposure effects – future studies could apply touch already during the first presentation of each stimulus. Based on the high memory performance of our participants, it is possible that introducing the touch manipulation only during re-exposure (t2/t3) limited its impact on memory, as the faces may have already been strongly encoded during the initial presentation without touch. In addition, varying the retention interval may yield different outcomes, as shorter or longer delays than the two-day interval used in the present study could differentially affect the potential influence of touch on memory processes. Given the limited social embeddedness of our experimental design, more ecologically valid approaches, such as field experiments involving naturalistic social interactions or studies exploring the impact of different levels of familiarity with the touching person, will be necessary to draw more nuanced conclusions about the potential absence (or presence) of touch effects on social memory. Additionally, the effects of longer social touch interventions, such as massages, or of actively performing touch compared to passively receiving it, on memory warrant examination. Given the immense relevance of faces in our day-to-day lives^65^, touch effects should also be tested through real-life ecological momentary assessments.

## Conclusion

Our findings provide moderate to strong evidence that receiving brief social touch during encoding in a controlled laboratory setting does not measurably influence face recognition memory. Evidence regarding metacognitive sensitivity and social evaluations was less consistent and in part inconclusive, although no significant touch effects were observed. Together, these results suggest that brief social touch in a controlled laboratory context has limited measurable impact on face memory and related evaluations, highlighting the likely importance of contextual factors such as meaningful social relevance or interpersonal interaction in shaping the influence of social touch.

## Supplementary Information

Supplementary Information.

## Data Availability

All study materials, primary data, and analysis scripts are publicly available (10.17605/OSF.IO/TZRFX). Face images were taken from the Oslo Face database (https://affectivebrains.com/oslo-face-database/).

## Declarations

### Generative AI and AI-assisted technologies in the writing process

The authors did not use generative AI technologies or AI-assisted tools for the preparation of this work.

## References

[1] Cascio, C. J., Moore, D. & McGlone, F. Social touch and human development. *Dev. Cogn. Neurosci.* **35**, 5–11 (2019). 29731417 10.1016/j.dcn.2018.04.009 PMC6968965

[2] Suvilehto, J. T., Cekaite, A. & Morrison, I. The why, who and how of social touch. *Nat. Rev. Psychol.* **2**, 606–621 (2023).

[3] Duhn, L. The importance of touch in the development of attachment. *Adv. Neonatal Care.* **10**, 294–300 (2010). 21102171 10.1097/ANC.0b013e3181fd2263

[4] Dunbar, R. I. M. The social role of touch in humans and primates: Behavioural function and neurobiological mechanisms. *Neurosci. Biobehav. Rev.* **34**, 260–268 (2010). 18662717 10.1016/j.neubiorev.2008.07.001

[5] Dueren, A. L. et al. Perspectives on interpersonal touch are related to subjective sleep quality. *J. Sleep Res.* **32**, e13766 (2023). 36351704 10.1111/jsr.13766 PMC10909536

[6] Eckstein, M., Mamaev, I., Ditzen, B. & Sailer, U. Calming effects of touch in human, animal, and robotic interaction: Scientific state-of-the-art and technical advances. *Front. Psychiatry.* **11**, 555058 (2020). 33329093 10.3389/fpsyt.2020.555058 PMC7672023

[7] Morrison, I. Keep calm and cuddle on: Social touch as a stress buffer. *Adapt. Hum. Behav. Physiol.* **2**, 344–362 (2016).

[8] Shamay-Tsoory, S. G. & Eisenberger, N. I. Getting in touch: A neural model of comforting touch. *Neurosci. Biobehav. Rev.* **130**, 263–273 (2021). 34474048 10.1016/j.neubiorev.2021.08.030

[9] Packheiser, J. et al. A systematic review and multivariate meta-analysis of the physical and mental health benefits of touch interventions. *Nat. Hum. Behav.* **8**, 1088–1107 (2024). 38589702 10.1038/s41562-024-01841-8 PMC11199149

[10] Hertenstein, M. J., Keltner, D., App, B., Bulleit, B. A. & Jaskolka, A. R. Touch communicates distinct emotions. *Emot. Wash. DC* **6**, 528–533 (2006). 10.1037/1528-3542.6.3.528 16938094

[11] McIntyre, S. et al. The language of social touch is intuitive and quantifiable. *Psychol. Sci.* **33**, 1477–1494 (2022). 35942875 10.1177/09567976211059801

[12] McGlone, F., Wessberg, J. & Olausson, H. Discriminative and affective touch: Sensing and feeling. *Neuron* **82**, 737–755 (2014). 24853935 10.1016/j.neuron.2014.05.001

[13] Olausson, H., Wessberg, J., Morrison, I., McGlone, F. & Vallbo, Å. The neurophysiology of unmyelinated tactile afferents. *Neurosci. Biobehav. Rev.* **34**, 185–191 (2010). 18952123 10.1016/j.neubiorev.2008.09.011

[14] Olausson, H. et al. Unmyelinated tactile afferents signal touch and project to insular cortex. *Nat. Neurosci.* **5**, 900–904 (2002). 12145636 10.1038/nn896

[15] Löken, L. S., Wessberg, J., Morrison, I., McGlone, F. & Olausson, H. Coding of pleasant touch by unmyelinated afferents in humans. *Nat. Neurosci.* **12**, 547–548 (2009). 19363489 10.1038/nn.2312

[16] Croy, I. et al. Interpersonal stroking touch is targeted to C tactile afferent activation. *Behav. Brain Res.* **297**, 37–40 (2016). 26433145 10.1016/j.bbr.2015.09.038

[17] Crusco, A. H. & Wetzel, C. G. The Midas touch: The effects of interpersonal touch on restaurant tipping. *Pers. Soc. Psychol. Bull.* **10**, 512–517 (1984).

[18] Fisher, J. D., Rytting, M. & Heslin, R. Hands touching hands: Affective and evaluative effects of an interpersonal touch. *Sociometry* **39**, 416–421 (1976). 1006362

[19] Schirmer, A., Wijaya, M. T. & Liu, S. The Midas effect: How somatosensory impressions shape affect and other-concern. In *Affective Touch and the Neurophysiology of CT Afferents* (eds Olausson, H. et al.) 283–299 (Springer New York, 2016). 10.1007/978-1-4939-6418-5_17.

[20] Schaefer, M., Kühnel, A., Rumpel, F. & Gärtner, M. Altruistic acting caused by a touching hand: Neural underpinnings of the Midas touch effect. *Soc. Cogn. Affect. Neurosci.* **17**, 437–446 (2022). 34746947 10.1093/scan/nsab119 PMC9071415

[21] Wingenbach, T. S. H., Ribeiro, B., Nakao, C., Gruber, J. & Boggio, P. S. Evaluations of affective stimuli modulated by another person’s presence and affiliative touch. *Emotion* **21**, 360–375 (2021). 31724416 10.1037/emo0000700

[22] Pawling, R., Trotter, P. D., McGlone, F. P. & Walker, S. C. A positive touch: C-tactile afferent targeted skin stimulation carries an appetitive motivational value. *Biol. Psychol.* **129**, 186–194 (2017). 28865933 10.1016/j.biopsycho.2017.08.057

[23] Schirmer, A., Ng, T. & Ebstein, R. P. Vicarious social touch biases gazing at faces and facial emotions. *Emotion* **18**, 1097–1105 (2018). 29389206 10.1037/emo0000393

[24] Spapé, M. M., Harjunen, V. & Ravaja, N. Effects of touch on emotional face processing: A study of event-related potentials, facial EMG and cardiac activity. *Biol. Psychol.* **124**, 1–10 (2017). 28089714 10.1016/j.biopsycho.2017.01.002

[25] Ellingsen, D.-M. et al. In touch with your emotions: oxytocin and touch change social impressions while others’ facial expressions can alter touch. *Psychoneuroendocrinology* **39**, 11–20 (2014). 24275000 10.1016/j.psyneuen.2013.09.017

[26] Chu, S. & Downes, J. J. Odour-evoked autobiographical memories: Psychological investigations of Proustian phenomena. *Chem. Senses* **25**, 111–116 (2000). 10668001 10.1093/chemse/25.1.111

[27] Mado Proverbio, A. et al. The effect of background music on episodic memory and autonomic responses: listening to emotionally touching music enhances facial memory capacity. *Sci. Rep.* **5**, 15219 (2015). 26469712 10.1038/srep15219 PMC4606564

[28] Matusz, P. J., Wallace, M. T. & Murray, M. M. A multisensory perspective on object memory. *Neuropsychologia* **105**, 243–252 (2017). 28400327 10.1016/j.neuropsychologia.2017.04.008 PMC5632572

[29] Ramot, M., Walsh, C. & Martin, A. Multifaceted integration: Memory for faces is subserved by widespread connections between visual, memory, auditory, and social networks. *J. Neurosci.* **39**, 4976–4985 (2019). 31036762 10.1523/JNEUROSCI.0217-19.2019 PMC6670243

[30] Schindler, S., Vormbrock, R. & Kissler, J. Encoding in a social feedback context enhances and biases behavioral and electrophysiological correlates of long-term recognition memory. *Sci. Rep.* **12**, 3312 (2022). 35228604 10.1038/s41598-022-07270-9 PMC8885702

[31] Eskenazi, T., Doerrfeld, A., Logan, G. D., Knoblich, G. & Sebanz, N. Your words are my words: Effects of acting together on encoding. *Q. J. Exp. Psychol.* **66**, 1026–1034 (2013). 10.1080/17470218.2012.725058 23035698

[32] Wagner, U., Schlechter, P. & Echterhoff, G. Socially induced false memories in the absence of misinformation. *Sci. Rep.* **12**, 7725 (2022). 35545651 10.1038/s41598-022-11749-w PMC9095591

[33] Perini, I., Morrison, I. & Olausson, H. Seeking pleasant touch: neural correlates of behavioral preferences for skin stroking. *Front. Behav. Neurosci.* **9**, 8 (2015). 25698948 10.3389/fnbeh.2015.00008 PMC4318429

[34] Talarico, J. M., Berntsen, D. & Rubin, D. C. Positive emotions enhance recall of peripheral details. *Cogn. Emot.* **23**, 380–398 (2009). 21359127 10.1080/02699930801993999 PMC3044328

[35] Madan, C. R., Scott, S. M. E. & Kensinger, E. A. Positive emotion enhances association-memory. *Emotion* **19**, 733–740 (2019). 30124317 10.1037/emo0000465 PMC6612425

[36] Lydon, J. & Karremans, J. C. Relationship regulation in the face of eye candy: A motivated cognition framework for understanding responses to attractive alternatives. *Curr. Opin. Psychol.* **1**, 76–80 (2015).

[37] Scheele, D. et al. Oxytocin modulates social distance between males and females. *J. Neurosci.* **32**, 16074–16079 (2012). 23152592 10.1523/JNEUROSCI.2755-12.2012 PMC6794013

[38] Green, P. & MacLeod, C. J. simr: An R package for power analysis of generalised linear mixed models by simulation. *Methods Ecol. Evol.* **7**, 493–498 (2016).

[39] Arend, M. G. & Schäfer, T. Statistical power in two-level models: A tutorial based on Monte Carlo simulation. *Psychol. Methods* **24**, 1–19 (2019). 30265048 10.1037/met0000195

[40] Russell, R., Duchaine, B. & Nakayama, K. Super-recognizers: People with extraordinary face recognition ability. *Psychon. Bull. Rev.* **16**, 252–257 (2009). 19293090 10.3758/PBR.16.2.252 PMC3904192

[41] Arrington, M., Elbich, D., Dai, J., Duchaine, B. & Scherf, K. S. Introducing the female Cambridge face memory test – Long form (F-CFMT+). *Behav. Res. Methods* **54**, 3071–3084 (2022). 35194750 10.3758/s13428-022-01805-8 PMC8863095

[42] Chelnokova, O. et al. Rewards of beauty: The opioid system mediates social motivation in humans. *Mol. Psychiatry* **19**, 746–747 (2014). 24514570 10.1038/mp.2014.1

[43] Gazzola, V. et al. Primary somatosensory cortex discriminates affective significance in social touch. *Proc. Natl. Acad. Sci.*10.1073/pnas.1113211109 (2012). 22665808 10.1073/pnas.1113211109 PMC3382530

[44] Suvilehto, J. T., Glerean, E., Dunbar, R. I. M., Hari, R. & Nummenmaa, L. Topography of social touching depends on emotional bonds between humans. *Proc. Natl. Acad. Sci. U.S.A.* **112**, 13811–13816 (2015). 26504228 10.1073/pnas.1519231112 PMC4653180

[45] Suvilehto, J. T. et al. Cross-cultural similarity in relationship-specific social touching. *Proc. R. Soc. B Biol. Sci.* **286**, 20190467 (2019). 10.1098/rspb.2019.0467 PMC6501924 31014213

[46] Fleming, S. M. & Lau, H. C. How to measure metacognition. *Front. Hum. Neurosci.* **8**, 443 (2014). 25076880 10.3389/fnhum.2014.00443 PMC4097944

[47] Maniscalco, B. & Lau, H. A signal detection theoretic approach for estimating metacognitive sensitivity from confidence ratings. *Conscious. Cogn.* **21**, 422–430 (2012). 22071269 10.1016/j.concog.2011.09.021

[48] Lapp, H. & Croy, I. Insights from the German version of the social touch questionnaire: How attitude towards social touch relates to symptoms of social anxiety. *Neuroscience* **464**, 133–142 (2020). 32673628 10.1016/j.neuroscience.2020.07.012

[49] Rekers, S., Heine, J., Thöne-Otto, A. I. T. & Finke, C. Neuropsychiatric symptoms and metamemory across the life span: Psychometric properties of the German Multifactorial Memory Questionnaire (MMQ). *J. Neurol.* **271**, 4551–4565 (2024). 38717611 10.1007/s00415-024-12402-4 PMC11233313

[50] Jungmann, S. M., Becker, F. & Witthöft, M. Erfassung der Lebendigkeit mentaler Vorstellungsbilder. *Diagnostica*10.1026/0012-1924/a000291 (2022).

[51] Posit team. *RStudio: Integrated Development Environment for R* (Posit Software, PBC, 2025).

[52] R Core Team. *R: A Language and Environment for Statistical Computing* (R Foundation for Statistical Computing, 2024).

[53] Bates, D., Mächler, M., Bolker, B. & Walker, S. Fitting linear mixed-effects models using lme4. *J. Stat. Softw.* **67**, 1–48 (2015).

[54] Keysers, C., Gazzola, V. & Wagenmakers, E.-J. Using Bayes factor hypothesis testing in neuroscience to establish evidence of absence. *Nat. Neurosci.* **23**, 788–799 (2020). 32601411 10.1038/s41593-020-0660-4 PMC7610527

[55] Riesthuis, P., Mangiulli, I., Broers, N. & Otgaar, H. Expert opinions on the smallest effect size of interest in false memory research. *Appl. Cogn. Psychol.* **36**, 203–215 (2022).

[56] Convertino, G. et al. Positive and negative touch differentially modulate metacognitive memory judgements for emotional stimuli. *Br. J. Psychol.* **116**, 34–51 (2025). 39259183 10.1111/bjop.12733

[57] Rosenberger, L. A., Ree, A., Eisenegger, C. & Sailer, U. Slow touch targeting CT-fibres does not increase prosocial behaviour in economic laboratory tasks. *Sci. Rep.* **8**, 7700 (2018). 29769551 10.1038/s41598-018-25601-7 PMC5955966

[58] Scheele, D. et al. An Oxytocin-induced facilitation of neural and emotional responses to social touch correlates inversely with Autism traits. *Neuropsychopharmacology* **39**, 2078–2085 (2014). 24694924 10.1038/npp.2014.78 PMC4104346

[59] Kreuder, A.-K. et al. Oxytocin enhances the pain-relieving effects of social support in romantic couples. *Hum. Brain Mapp.* **40**, 242–251 (2019). 30152573 10.1002/hbm.24368 PMC6865468

[60] Valori, I., Jung, M. M. & Fairhurst, M. T. Social touch to build trust: A systematic review of technology-mediated and unmediated interactions. *Comput. Hum. Behav.* **153**, 108121 (2024).

[61] Della Longa, L., Gliga, T. & Farroni, T. Tune to touch: Affective touch enhances learning of face identity in 4-month-old infants. *Dev. Cogn. Neurosci.* **35**, 42–46 (2019). 29153656 10.1016/j.dcn.2017.11.002 PMC6347579

[62] Stevens, L., Bregulla, M. & Scheele, D. Out of touch? How trauma shapes the experience of social touch: Neural and endocrine pathways. *Neurosci. Biobehav. Rev.* **159**, 105595 (2024). 38373642 10.1016/j.neubiorev.2024.105595

[63] Voelter, J., Postin, D., Croy, I., Hurlemann, R. & Scheele, D. A neural signature of touch aversion and interpersonal problems in borderline personality disorder. *Psychother. Psychosom.*10.1159/000545973 (2025). 40349695 10.1159/000545973

[64] Smith, S. M. & Vela, E. Environmental context-dependent memory: A review and meta-analysis.. *Psychon. Bull. Rev.* **8**, 203–220 (2001). 11495110 10.3758/bf03196157

[65] Young, A. W. & Burton, A. M. Are we face experts?. *Trends Cogn. Sci.* **22**, 100–110 (2018). 29254899 10.1016/j.tics.2017.11.007

