# Supplementary material for: Affective touch and face recognition: effects on memory and metacognitive performance
Source: Sci Rep. 2026 Mar 31;16:10991. doi: 10.1038/s41598-026-43969-9 (PMC13043764; doi:10.1038/s41598-026-43969-9)
Supplement: Supplementary file 1 — Supplementary Information. [file 41598_2026_43969_MOESM1_ESM.docx]

Supplementary Information

# Additional information regarding experimental procedure

## Exact timeline of testing sessions

Participants were recruited through advertisements using flyers around the university campus, social media, as well as through advertisements on a study participation portal of the university between April 2024 and November 2024.

After signing up for the study, participants were sent a link to an online questionnaire (Sosci Survey) that asked them about each of the inclusion criteria for the study. Only participants who met all inclusion criteria according to their answers in that questionnaire were invited to take part in the actual experiment.

| Encoding | Retrieval (Approx. 48 hours later) |
| --- | --- |
| Introduction, study information, informed consent form | Introduction, further study information |
| Questionnaire for demographic information, mood (Sosci Survey) | Questionnaire with mood (Sosci Survey) |
| Cambridge face memory test male and female version (Presented via Testable) | Face recognition task (Sosci Survey) |
| Face encoding task (MatLab/Psychtoolbox) | emBODY Touch maps (Nummenmaa et al., 2014; Suvilehto et al., 2015) |
| Questionnaires: Beck Depression Inventory II, Liebowitz Social Anxiety Scale, Autism Questionnaire, Social Touch Questionnaire, Longing for Interpersonal Touch Questionnaire, UCLA Loneliness Scale (Sosci Survey) | Questionnaires: Plymouth Sensory Imagination Questionnaire, Meta Memory Questionnaire (Subscales Satisfaction and Ability), Childhood Trauma Questionnaire, Trauma History Questionnaire, |
|  | Debriefing: Research question and purpose of the study, information regarding course credit/monetary compensation |
| Duration: approx. 120 Minutes | Duration: Approx. 75 Minutes |

The exact study materials (in German), instructions (in German), as well as the code for the face encoding task can be found on [osf](https://osf.io/tzrfx/overview?view_only=a84f25e0fa9b4c69a6dd8c6176af9c54). The images for the face database [have to be requested](https://affectivebrains.com/oslo-face-database/) from the original authors of the database. The versions of the Cambridge Face Memory test have to be requested via the platform [Testable](https://www.testable.org/library) from the [original authors](https://lab.faceblind.org/test_index).

## Set-up of the testing room

The experimental sessions took place in a windowless testing room with constant lighting. The first and second experimental session took place in the same room.

Participants were seated in front of a computer screen (monitor size 27”). They used keyboard and mouse to navigate all parts of the experiment.

For the face encoding task, participants were instructed to place their left arm through a hole that was cut into a large curtain, that created a cubicle within which the experimenter who performed the touches remained hidden. They placed their arm on a foam block that was covered with a water-resistant tablecloth (to enable the pillow to be sanitized after each participant). Participants were instructed to move their arm and adjust the chair position before the start of the face encoding task to ensure they were comfortable enough to remain in the position throughout the task.

## Power analysis

We specified medium effect sizes of γ = .30 for the first level direct effects (touch condition), the second level direct effect (attitude towards social touch), and the cross-level interaction (touch condition * attitude towards social touch) as well as a medium effect size (τ = .09) for the random slope. We simulated both medium and large ICCs (ρ = .30 and .50). The power estimation for each second level sample size was based on 1000 simulations.

## Excluded participants

Three participants had to be excluded after the conclusion of data collection. Two participants reported during the demographics questionnaire that they were on psychopharmacological medication and had psychiatric illness, even though they had denied having any diagnoses or taking any medication in the screening questionnaire. One participant was excluded because they reported drug consumption (marijuana) on the day of the appointment.

## Data extraction

The raw questionnaire data from SosciSurvey was retrieved as Microsoft Excel file (.xlsx) for each of the questionnaires. Automatic additional metadata like IP Adress and timestamps were removed.

Logfiles for each participant were merged with responses of the recognition task using a Matlab script and merged with the questionnaire data.

Logdata for the CFMT+/fCFMT+ were saved as text files after completing the experiment on Testable. The information was read out with a Matlab script to generate scores for each participant which were then merged with the dataset.

The raw data for each participant, raw questionnaire data and data extraction scripts can be found on osf.

# Descriptive statistics

## Outcome parameters

Table 1. Descriptive statistics for the outcome parameters of the study.

| Variable | Mean | SD | Median | Range |
| --- | --- | --- | --- | --- |
| Correct responses | 77.02 | 14.02 | 79 | [41;96] |
| False positive responses | 2.42 | 2.99 | 1 | [0;12] |
| Correct rejections | 45.57 | 2.99 | 47 | [36;48] |
| False negative responses | 18.98 | 14.02 | 17 | [0;55] |
| d’ | 2.74 | 0.88 | 2.67 | [1.11;4.48] |
| Confidence ratings | 8.43 | 1.10 | 8.61 | [5.74;10] |
| Meta d’ | 2.01 | 1.00 | 1.87 | [0.35;4.42] |
| Attractiveness rating (t1) | 4.65 | 1.33 | 4.69 | [1.31;6.94] |
| Trustworthiness rating (t1) | 5.53 | 1.24 | 5.63 | [2.88;8.66] |
| Attractiveness rating (mean(t2,t3)-t1) | -0.06 | 0.39 | 0.00 | [-1.41;0.66] |
| Trustworthiness rating (mean(t2,t3)-t1) | -0.23 | 0.34 | -0.19 | [-1.22;0.56] |

## Predictor variables

Table 2. Descriptive statistics for the predictor/control variables of the study.

| Variable | Mean | SD | Median | Range |
| --- | --- | --- | --- | --- |
| Participant age | 27.13 | 8.16 | 25 | [19;61] |
| STQ-Score | 38.39 | 11.78 | 39 | [12;66] |
| MMQ-Ability | 52.58 | 13.36 | 52 | [10;75] |
| MMQ-Satisfaction | 27.05 | 6.76 | 25 | [17;45] |
| PSI-Q | 7.50 | 1.55 | 7.76 | [4;10] |
| CFMT+ | 68.41 | 11.63 | 68 | [41;93] |
| fCFMT+ | 80.35 | 11.15 | 83 | [54;98] |
| Touch pleasantness (static) | 3.55 | 0.95 | 3.5 | [1;5] |
| Touch pleasantness (affective) | 3.43 | 1.08 | 3.5 | [1;5] |
| Note. STQ = Social Touch Questionnaire, MMQ = Meta Memory Questionnaire, PSI-Q = Plymouth Sensory Imagination Questionnaire, CFMT+ = Cambridge Face Memory Test Long Version, fCFMT+ = female Cambridge Face Memory Test Long Version | | | | |

# Model parameters

## Recognition accuracy

### Touch versus no touch

Table 3. Full models for d' (touch versus no touch). Models without random slope.

|  | Bayesian (Prior: small effect) | | | Bayesian (Prior: medium effect) | | | Bayesian (Prior: large effect) | | | Frequentist | | | |
| --- | --- | --- | --- | --- | --- | --- | --- | --- | --- | --- | --- | --- | --- |
|  | Beta | SE | CI | Beta | SE | CI | Beta | SE | CI | Beta | T(df) | p | CI |
| Intercept | 2.78 | 0.11 | [2.57;3.00] | 2.78 | 0.11 | [2.56;2.99] | 2.78 | 0.11 | [2.56;3.00] | 2.78 | 25.36(54) | <.001 | [2.58;3.00] |
| Condition (Touch) | 0.01 | 0.05 | [-0.08;0.10] | 0.01 | 0.05 | [-0.08;0.10] | 0.01 | 0.05 | [-0.08;0.09] | 0.01 | 0.21(54) | 0.84 | [-0.08;0.10] |
| Sex (male) | -0.34 | 0.21 | [-0.76;0.05] | -0.35 | 0.21 | [-0.78;0.06] | -0.34 | 0.21 | [-0.76;0.09] | -0.34 | -1.65(47) | 0.11 | [-0.76;0.08] |
| PSI-Q | -0.07 | 0.07 | [-0.20; 0.06] | -0.08 | 0.07 | [-0.21; 0.06] | -0.07 | 0.07 | [-0.20; 0.06] | -0.07 | -1.07(47) | 0.29 | [-0.20;0.06] |
| MMQ Ability | 0.01 | 0.01 | [-0.01; 0.03] | 0.01 | 0.01 | [-0.01; 0.03] | 0.01 | 0.01 | [-0.01; 0.03] | 0.01 | 0.69(47) | 0.49 | [-0.01;0.03] |
| MMQ Satisfaction | 0.00 | 0.02 | [-0.04; 0.04] | 0.00 | 0.02 | [-0.04; 0.04] | 0.00 | 0.02 | [-0.04; 0.04] | 0.00 | 0.15(47) | 0.89 | [-0.04; 0.04] |
| CFMT+ | 0.02 | 0.01 | [0.00; 0.05] | 0.02 | 0.01 | [0.00; 0.05] | 0.02 | 0.01 | [0.00; 0.04] | 0.02 | 2.21(47) | 0.03 | [0.00;0.05] |
| fCFMT+ | 0.03 | 0.01 | [0.00; 0.05] | 0.03 | 0.01 | [0.00; 0.05] | 0.03 | 0.01 | [0.00; 0.05] | 0.03 | 2.27(47) | 0.03 | [0.00;0.05] |
| Age | 0.01 | 0.01 | [-0.02; 0.03] | 0.01 | 0.01 | [-0.02; 0.03] | 0.00 | 0.03 | [-0.02; 0.03] | 0.01 | 0.40(47) | 0.69 | [-0.02; 0.03] |

Table 4. Model fit (stepwise approach) for d’ (touch versus no touch)

|  | df | AIC | BIC | Likelihood ratio |
| --- | --- | --- | --- | --- |
| Empty model + covariates | 10 | 199.31 | 225.56 | / |
| Empty model + covariates + condition | 11 | 205.67 | 234.43 | 4.36, p = .04 |
| Empty model + covariates + condition + random slope | 13 | 208.03 | 242.03 | 1.63, p = 0.44 |

Table 5. Bayes factors (touch versus no touch)

|  | Prior: small effect | Prior: medium effect | Prior: large effect |
| --- | --- | --- | --- |
| BF_10_ | 8.26 | 9.15 | 8.30 |

### Static versus dynamic versus no touch

Table 6. Full models for d' (static versus dynamic versus no touch). Models without random slope.

|  | Bayesian (Prior: small effect) | | | Bayesian (Prior: medium effect) | | | Bayesian (Prior: large effect) | | | Frequentist | | | |
| --- | --- | --- | --- | --- | --- | --- | --- | --- | --- | --- | --- | --- | --- |
|  | Beta | SE | CI | Beta | SE | CI | Beta | SE | CI | Beta | T(df) | p | CI |
| Intercept | 2.77 | 0.11 | [2.55;2.99] | 2.78 | 0.11 | [2.55;3.00] | 2.78 | 0.11 | [2.54;3.00] | 2.77 | 24.62(108) | <.001 | [2.55;3.00] |
| Condition (Dynamic Touch) | -0.01 | 0.05 | [-0.12;0.09] | -0.01 | 0.05 | [-0.12;0.09] | -0.01 | 0.05 | [-0.12;0.10] | -0.01 | -0.20  (108) | 0.84 | [-0.12;0.10] |
| Condition (Static Touch) | 0.01 | 0.05 | [-0.10;0.11] | 0.01 | 0.05 | [-0.10;0.12] | 0.01 | 0.05 | [-0.10;0.11] | 0.01 | 0.13  (108) | 0.89 | [-0.10;0.11] |
| Sex (male) | -0.32 | 0.22 | [-0.73;0.11] | -0.32 | 0.22 | [-0.75;0.09] | -0.33 | 0.21 | [-0.73;0.07] | -0.32 | -1.53  (47) | 0.13 | [-0.75;0.10] |
| PSI-Q | -0.07 | 0.07 | [-0.20;0.06] | -0.07 | 0.07 | [-0.21;0.07] | -0.07 | 0.07 | [-0.21;0.06] | -0.07 | -1.02  (47) | 0.31 | [-0.20;0.07] |
| MMQ Ability | 0.01 | 0.01 | [-0.01;0.03] | 0.01 | 0.01 | [-0.02;0.03] | 0.01 | 0.01 | [-0.01;0.03] | 0.01 | 0.57  (47) | 0.57 | [-0.01;0.03] |
| MMQ Satisfaction | 0.00 | 0.02 | [-0.04;0.04] | 0.00 | 0.02 | [-0.04;0.04] | 0.00 | 0.02 | [-0.03;0.04] | 0.00 | 0.09  (47) | 0.93 | [-0.04;0.04] |
| CFMT+ | 0.02 | 0.01 | [0.00;0.05] | 0.02 | 0.01 | [0.00;0.05] | 0.02 | 0.01 | [0.00;0.05] | 0.02 | 2.17  (47) | 0.04 | [0.00;0.05] |
| fCFMT+ | 0.03 | 0.01 | [0.00;0.05] | 0.03 | 0.01 | [0.00;0.05] | 0.03 | 0.01 | [0.00;0.05] | 0.03 | 2.22  (47) | 0.03 | [0.00;0.05] |
| Age | 0.00 | 0.01 | [-0.02;0.03] | 0.01 | 0.01 | [-0.02;0.03] | 0.01 | 0.01 | [-0.02;0.03] | 0.00 | 0.34  (47) | 0.73 | [-0.02;0.03] |

Table 7. Model fit for stepwise approach

|  | df | AIC | BIC | Likelihood ratio |
| --- | --- | --- | --- | --- |
| Empty model + covariates | 10 | 262.10 | 292.66 | / |
| Empty model + covariates + condition | 12 | 274.26 | 310.78 | 8.16, p = 0.01 |
| Empty model + covariates + condition + random slope | 17 | 279.60 | 331.34 | 4.66, p = 0.46 |

Table 8. Bayes factors for d' static versus dynamic versus no touch)

|  | Prior: small effect | Prior: medium effect | Prior: large effect |
| --- | --- | --- | --- |
| BF | 53.93 | 54.60 | 60.91 |

## Confidence Ratings

### Touch versus no touch

Table 9. Full models for confidence ratings (touch vs no touch). Models without random slope.

|  | Bayesian (Prior: small effect) | | | Bayesian (Prior: medium effect) | | | Bayesian (Prior: large effect) | | | Frequentist | | | |
| --- | --- | --- | --- | --- | --- | --- | --- | --- | --- | --- | --- | --- | --- |
|  | Beta | SE | CI | Beta | SE | CI | Beta | SE | CI | Beta | T(df) | p | CI |
| Intercept | 8.31 | 0.17 | [7.98;8.63] | 8.32 | 0.17 | [7.98;8.67] | 8.32 | 0.17 | [7.99;8.66] | 8.32 | 49.84(54) | <.001 | [7.98;8.65] |
| Condition (Touch) | 0.03 | 0.05 | [-0.07;0.13] | 0.03 | 0.05 | [-0.08;0.13] | 0.03 | 0.05 | [-0.08;0.13] | 0.03 | 0.57(54) | 0.57 | [-0.07;0.13] |
| Sex (male) | 0.18 | 0.31 | [-0.46;0.79] | 0.18 | 0.32 | [-0.44;0.80] | 0.18 | 0.33 | [-0.49;0.82] | 0.18 | 0.57(47) | 0.57 | [-0.46;0.83] |
| PSI-Q | 0.19 | 0.10 | [-0.03;0.39] | 0.19 | 0.10 | [-0.02;0.39] | 0.19 | 0.10 | [-0.01;0.39] | 0.19 | 1.85(47) | 0.19 | [-0.02, 0.39] |
| MMQ Ability | 0.00 | 0.02 | [-0.03;0.03] | 0.00 | 0.02 | [-0.03;0.03] | 0.00 | 0.02 | [-0.03;0.03] | 0.01 | 0.04(47) | 0.97 | [-0.03;0.03] |
| MMQ Satisfaction | 0.01 | 0.03 | [-0.04;0.07] | 0.01 | 0.03 | [-0.05;0.07] | 0.01 | 0.03 | [-0.05;0.07] | 0.01 | 0.41(47) | 0.68 | [-0.05;0.07] |
| CFMT+ | 0.02 | 0.02 | [-0.01;0.06] | 0.02 | 0.02 | [-0.01;0.06] | 0.02 | 0.02 | [-0.01;0.06] | 0.02 | 1.42(47) | 0.16 | [-0.01;0.06] |
| fCFMT+ | 0.02 | 0.02 | [-0.01;0.06] | 0.02 | 0.02 | [-0.01;0.06] | 0.02 | 0.02 | [-0.01;0.06] | 0.02 | 1.28(47) | 0. 21 | [-0.01;0.06] |
| Age | 0.01 | 0.02 | [-0.03;0.04] | 0.01 | 0.02 | [-0.04;0.05] | 0.01 | 0.02 | [-0.04;0.05] | 0.01 | 0.25(47) | 0.81 | [-0.04;0.05] |

Table 10. Model fits for stepwise approach

|  | df | AIC | BIC | Likelihood Ratio |
| --- | --- | --- | --- | --- |
| Empty model + covariates | 10 | 254.20 | 280.45 | / |
| Empty model + covariates + condition | 11 | 260.01 | 288.78 | 3.81, p = .05 |
| Empty model + covariates + condition + random slope | 13 | 262.76 | 296.76 | 1.25, p = .54 |

Table 11. Bayes factors for confidence ratings (touch versus no touch)

|  | Prior: small effect | Prior: medium effect | Prior: large effect |
| --- | --- | --- | --- |
| BF | 7.20 | 6.98 | 7.21 |

### Static versus dynamic versus no touch

Full model

Table 12. Full models for confidence ratings (static versus dynamic versus no touch) with no random slope.

|  | Bayesian (Prior: small effect) | | | Bayesian (Prior: medium effect) | | | Bayesian (Prior: large effect) | | | Frequentist | | | |
| --- | --- | --- | --- | --- | --- | --- | --- | --- | --- | --- | --- | --- | --- |
|  | Beta | SE | CI | Beta | SE | CI | Beta | SE | CI | Beta | T(df) | p | CI |
| Intercept | 8.32 | 0.17 | [7.97;8.64] | 8.30 | 0.17 | [7.96;8.64] | 8.32 | 0.17 | [7.97;8.65] | 8.31 | 49.47(108) | <0.001 | [7.98;8.64] |
| Condition (Dynamic Touch) | 0.03 | 0.06 | [-0.09;0.16] | 0.04 | 0.07 | [-0.09;0.17] | 0.04 | 0.07 | [-0.09;0.17] | 0.04 | 0.54(108) | 0.54 | [-0.09;0.16] |
| Condition (Static Touch) | 0.02 | 0.07 | [-0.11;0.15] | 0.02 | 0.07 | [-0.12;0.16] | 0.02 | 0.07 | [-0.11;0.15] | 0.02 | 0.34(108) | 0.34 | [-0.11;0.15] |
| Sex (male) | 0.20 | 0.32 | [-0.43;0.83] | 0.21 | 0.32 | [-0.43;0.83] | 0.21 | 0.32 | [-0.40;0.85] | 0.20 | 0.64(47) | 0.64 | [-0.44;0.84] |
| PSI-Q | 0.18 | 0.10 | [-0.03;0.37] | 0.18 | 0.10 | [-0.02;0.38] | 0.18 | 0.10 | [-0.02;0.38] | 0.18 | 1.75(47) | 0.09 | [-0.03;0.38] |
| MMQ Ability | 0.00 | 0.02 | [-0.03;0.03] | 0.00 | 0.01 | [-0.03;0.03] | 0.00 | 0.02 | [-0.03;0.03] | 0.00 | 0.15(47) | 0.89 | [-0.03,0.03] |
| MMQ Satisfaction | 0.01 | 0.03 | [-0.05;0.07] | 0.01 | 0.03 | [-0.04;0.07] | 0.01 | 0.03 | [-0.04;0.07] | 0.01 | 0.46(47) | 0.65 | [-0.05;0.07] |
| CFMT+ | 0.02 | 0.02 | [-0.01;0.06] | 0.02 | 0.02 | [-0.01;0.05] | 0.02 | 0.02 | [-0.01;0.06] | 0.02 | 1.29(47) | 0.20 | [-0.01;0.06] |
| fCFMT+ | 0.03 | 0.02 | [-0.01;0.06] | 0.02 | 0.02 | [-0.01;0.06] | 0.03 | 0.02 | [-0.01;0.06] | 0.03 | 1.45(47) | 0.15 | [-0.01;0.06] |
| Age | 0.01 | 0.02 | [-0.03;0.05] | 0.01 | 0.02 | [-0.03;0.05] | 0.01 | 0.02 | [-0.03;0.05] | 0.01 | 0.30(47) | 0.77 | [-0.04,0.05] |

Table 13. Model fits for stepwise approach

|  | df | AIC | BIC | Likelihood Ratio |
| --- | --- | --- | --- | --- |
| Empty model + covariates | 10 | 340.79 | 371.36 | / |
| Empty model + covariates + condition | 12 | 352.04 | 388.56 | 7.25, p = .03 |
| Empty model + covariates + condition + random slope | 17 | 357.27 | 409.00 | 4.77, p = .44 |

Table 14. Bayes factors for confidence ratings (static versus dynamic versus no touch)

|  | Prior: small effect | Prior: medium effect | Prior: large effect |
| --- | --- | --- | --- |
| BF | 38.12 | 37.42 | 37.27 |

## Metacognitive Sensitivity

### Touch versus no touch

Table 15. Full models for meta d' (touch versus no touch) with no random slope.

|  | Bayesian (Prior: small effect) | | | Bayesian (Prior: medium effect) | | | Bayesian (Prior: large effect) | | | Frequentist | | | |
| --- | --- | --- | --- | --- | --- | --- | --- | --- | --- | --- | --- | --- | --- |
|  | Beta | SE | CI | Beta | SE | CI | Beta | SE | CI | Beta | T(df) | p | CI |
| Intercept | 2.24 | 0.15 | [1.95;2.54] | 2.23 | 0.15 | [1.94;2.53] | 2.23 | 0.15 | [1.92;2.53] | 2.24 | 14.96(54) | <.001 | [1.94;2.54] |
| Condition (Touch) | -0.07 | 0.13 | [-0.32;0.19] | -0.06 | 0.13 | [-0.31;0.19] | -0.07 | 0.13 | [-0.32;0.19] | -0.07 | -0.52(54) | 0.60 | [-0.32;0.19] |
| Sex (male) | -0.39 | 0.27 | [-0.94;0.12] | -0.38 | 0.26 | [-0.90;0.13] | -0.29 | 0.26 | [-0.90;0.13] | -0.38 | -1.46(47) | 0.15 | [-0.92;0.15] |
| PSI-Q | -0.08 | 0.08 | [-0.25;0.09] | -0.08 | 0.08 | [-0.24;0.09] | -0.08 | 0.09 | [-0.24;0.09] | -0.08 | -0.08(47) | 0.36 | [-0.25;0.09] |
| MMQ Ability | 0.02 | 0.01 | [-0.01;0.04] | 0.02 | 0.01 | [-0.01;0.04] | 0.02 | 0.01 | [-0.01;0.04] | 0.02 | 1.26(47) | 0.22 | [-0.01;0.04] |
| MMQ Satisfaction | 0.01 | 0.02 | [-0.04;0.06] | 0.01 | 0.02 | [-0.04;0.06] | 0.01 | 0.02 | [-0.04;0.06] | 0.01 | 0.40(47) | 0.69 | [-0.04;0.06] |
| CFMT+ | 0.02 | 0.01 | [-0.01;0.04] | 0.02 | 0.01 | [-0.01;0.04] | 0.02 | 0.01 | [-0.01;0.04] | 0.02 | 1.11(47) | 0.27 | [-0.01;0.04] |
| fCFMT+ | 0.04 | 0.01 | [0.01;0.07] | 0.04 | 0.01 | [0.01;0.07] | 0.04 | 0.01 | [0.01;0.07] | 0.04 | 2.51(47) | 0.02 | [0.01;0.07] |
| Age | 0.02 | 0.02 | [-0.01;0.06] | 0.02 | 0.02 | [-0.01;0.05] | 0.02 | 0.02 | [-0.01;0.06] | 0.02 | 1.27(47) | 0.21 | [-0.01;0.06] |

Table 16. Model fits for stepwise approach.

|  | df | AIC | BIC | Likelihood Ratio |
| --- | --- | --- | --- | --- |
| Empty model + covariates | 10 | 335.57 | 361.82 |  |
| Empty model + covariates + condition | 11 | 339.62 | 368.39 | 2.05, p = 0.15 |
| Empty model + covariates + condition + random slope | 13 | 342.57 | 376.58 | 1.05, p = 0.59 |

Table 17. Bayes factors for meta d' (touch versus no touch).

|  | Prior: small effect | Prior: medium effect | Prior: large effect |
| --- | --- | --- | --- |
| BF | 2.86 | 2.69 | 2.84 |

### Static versus dynamic versus no touch

Table 18. Model fits for meta d' (static versus dynamic versus no touch) without random slope.

|  | Bayesian (Prior: small effect) | | | Bayesian (Prior: medium effect) | | | Bayesian (Prior: large effect) | | | Frequentist | | | |
| --- | --- | --- | --- | --- | --- | --- | --- | --- | --- | --- | --- | --- | --- |
|  | Beta | SE | CI | Beta | SE | CI | Beta | SE | CI | Beta | T(df) | p | CI |
| Intercept | 2.24 | 0.16 | [1.91;2.54] | 2.24 | 0.15 | [1.94;2.54] | 2.25 | 0.16 | [1.94;2.55] | 2.24 | 14.47(108) | <.001 | [1.94; 2.55] |
| Condition (Dynamic Touch) | 0.01 | 0.12 | [-0.23;0.24] | 0.01 | 0.12 | [-0.23;0.24] | 0.01 | 0.12 | [-0.21;0.24] | 0.01 | 0.06(108) | 0.95 | [-0.22;0.24] |
| Condition (Static Touch) | -0.06 | 0.12 | [-0.29;0.17] | -0.06 | 0.12 | [-0.29;0.17] | -0.06 | 0.12 | [-0.29;0.17] | -0.06 | -0.57(108) | 0.57 | [-0.29;0.16] |
| Sex (male) | -0.40 | 0.27 | [-0.93;0.15] | -0.39 | 0.27 | [-0.92;0.12] | -0.40 | 0.27 | [-0.95;0.14] | -0.40 | -1.45(47) | 0.15 | [-0.94;0.15] |
| PSI-Q | -0.07 | 0.09 | [-0.24;0.10] | -0.07 | 0.09 | [-0.25;0.10] | -0.07 | 0.09 | [-0.25;0.10] | -0.07 | -0.85(47) | 0.40 | [-0.25;0.10] |
| MMQ Ability | 0.01 | 0.01 | [-0.01;0.04] | 0.01 | 0.01 | [-0.01;0.04] | 0.01 | 0.01 | [-0.01;0.04] | 0.01 | 1.04(47) | 0.31 | [-0.01;0.04] |
| MMQ Satisfaction | 0.01 | 0.03 | [-0.04;0.06] | 0.01 | 0.03 | [-0.04;0.06] | 0.01 | 0.03 | [-0.04;0.06] | 0.01 | 0.47(47) | 0.64 | [-0.04;0.06] |
| CFMT+ | 0.02 | 0.01 | [-0.01;0.04] | 0.02 | 0.01 | [-0.01;0.04] | 0.02 | 0.01 | [-0.01;0.04] | 0.02 | 1.11(47) | 0.27 | [-0.01;0.04] |
| fCFMT+ | 0.04 | 0.02 | [0.01;0.07] | 0.04 | 0.01 | [0.01;0.07] | 0.04 | 0.02 | [0.01;0.07] | 0.04 | 2.48(47) | 0.02 | [0.01;0.07] |
| Age | 0.02 | 0.02 | [-0.01;0.06] | 0.02 | 0.02 | [-0.01;0.06] | 0.02 | 0.02 | [-0.01;0.06] | 0.02 | 1.33(47) | 0.19 | [-0.01;0.06] |

Table 19. Model fits for stepwise approach.

|  | df | AIC | BIC | Likelihood Ratio |
| --- | --- | --- | --- | --- |
| Empty model + covariates | 10 | 450.96 | 481.52 | / |
| Empty model + covariates + condition | 12 | 459.76 | 496.28 | 4.80, p = 0.09 |
| Empty model + covariates + condition + random slope | 17 | 460.18 | 511.92 | 9.58, p = 0.09 |

Table 20. Bayes factors for meta d' (static versus dynamic versus no touch).

|  | Prior: small effect | Prior: medium effect | Prior: large effect |
| --- | --- | --- | --- |
| BF | 11.64 | 10.54 | 10.79 |

## Moderation by STQ: Recognition Accuracy

### Touch versus no touch

Table 21. Full models for d' moderated by attitude towards social touch (touch versus no touch) without random slope.

|  | Bayesian (Prior: small effect) | | | Bayesian (Prior: medium effect) | | | Bayesian (Prior: large effect) | | | Frequentist | | | |
| --- | --- | --- | --- | --- | --- | --- | --- | --- | --- | --- | --- | --- | --- |
|  | Beta | SE | CI | Beta | SE | CI | Beta | SE | CI | Beta | T(df) | p | CI |
| Intercept | 2.79 | 0.11 | [2.58;3.01] | 2.78 | 0.11 | [2.56;3.00] | 2.79 | 0.11 | [2.57;3.02] | 2.79 | 25.14(53) | <.001 | [2.57;3.01] |
| Condition (Touch) | 0.01 | 0.05 | [-0.08;0.10] | 0.01 | 0.05 | [-0.08;0.10] | 0.01 | 0.05 | [-0.08;0.10] | 0.01 | 0.21(53) | 0.84 | [-0.08;0.10] |
| STQ | -0.01 | 0.01 | [-0.02;0.01] | -0.01 | 0.01 | [-0.02;0.01] | -0.01 | 0.01 | [-0.02;0.01] | 0.01 | -0.67(46) | 0.51 | [-0.32;0.16] |
| Sex (male) | -0.37 | 0.21 | [-0.79;0.05] | -0.36 | 0.21 | [-0.78;0.05] | -0.37 | 0.22 | [-0.81;0.06] | -0.37 | -1.72(46) | 0.09 | [-0.80;0.06] |
| PSI-Q | -0.08 | 0.07 | [-0.21; 0.06] | -0.08 | 0.07 | [-0.22; 0.06] | -0.08 | 0.07 | [-0.21; 0.06] | -0.08 | -1.13(46) | 0.27 | [-0.21;0.06] |
| MMQ Ability | 0.01 | 0.01 | [-0.01; 0.03] | 0.01 | 0.01 | [-0.01; 0.03] | 0.01 | 0.01 | [-0.02; 0.03] | 0.01 | 0.57(46) | 0.57 | [-0.02;0.03] |
| MMQ Satisfaction | 0.00 | 0.02 | [-0.03; 0.04] | 0.00 | 0.02 | [-0.03; 0.04] | 0.00 | 0.02 | [-0.04; 0.04] | 0.00 | 0.17(46) | 0.87 | [-0.04; 0.04] |
| CFMT+ | 0.02 | 0.01 | [0.00; 0.05] | 0.02 | 0.01 | [0.00; 0.05] | 0.02 | 0.01 | [0.00; 0.05] | 0.02 | 2.24(46) | 0.03 | [0.00;0.05] |
| fCFMT+ | 0.03 | 0.01 | [0.00; 0.05] | 0.03 | 0.01 | [0.00; 0.05] | 0.03 | 0.01 | [0.00; 0.05] | 0.03 | 2.20(46) | 0.03 | [0.00;0.05] |
| Age | 0.01 | 0.01 | [-0.02; 0.03] | 0.01 | 0.01 | [-0.02; 0.03] | 0.01 | 0.01 | [-0.02; 0.03] | 0.01 | 0.38(46) | 0.71 | [-0.02; 0.03] |
| Touch*STQ | 0.00 | 0.00 | [-0.01; 0.01] | 0.00 | 0.00 | [-0.01;0.01] | 0.00 | 0.00 | [-0.01;0.01] | 0.00 | 0.25(53) | 0.81 | [-0.01;0.01] |

Table 22. Model fit for stepwise approach.

|  | df | AIC | BIC | Likelihood ratio |
| --- | --- | --- | --- | --- |
| Empty model + covariates + condition + random slope | 13 | 208.03 | 242.03 | / |
| Empty model + covariates + condition + interaction + random slope | 15 | 228.57 | 267.50 | 16.54, p < 0.001 |

Table 23. Bayes factors for d' moderated by attitudes towards social touch (touch versus no touch)

|  | Prior: small effect | Prior: medium effect | Prior: large effect |
| --- | --- | --- | --- |
| BF | 3634.15 | 3671.33 | 3528.27 |

### Dynamic vs static vs no touch

Table 24. Full model for d' moderated by attitude towards social touch (static versus dynamic versus no touch).

|  | Bayesian (Prior: small effect) | | | Bayesian (Prior: medium effect) | | | Bayesian (Prior: large effect) | | | Frequentist | | | |
| --- | --- | --- | --- | --- | --- | --- | --- | --- | --- | --- | --- | --- | --- |
|  | Beta | SE | CI | Beta | SE | CI | Beta | SE | CI | Beta | T(df) | p | CI |
| Intercept | 2.78 | 0.12 | [2.55;3.01] | 2.78 | 0.12 | [2.55;3.02] | 2.78 | 0.12 | [2.55;3.00] | 2.78 | 24.42(106) | <.001 | [2.56;3.01] |
| Condition (Dynamic Touch) | -0.01 | 0.06 | [-0.12;0.10] | -0.01 | 0.06 | [-0.12;0.10] | -0.01 | 0.06 | [-0.12;0.10] | -0.01 | -0.21(106) | 0.84 | [-0.12;0.10] |
| Condition (Static Touch) | 0.01 | 0.06 | [-0.10;0.12] | 0.01 | 0.05 | [-0.10;0.12] | 0.01 | 0.06 | [-0.10;0.12] | 0.01 | 0.14(106) | 0.89 | [-0.10;0.12] |
| STQ | -0.01 | 0.01 | [-0.02;0.01] | -0.01 | 0.01 | [-0.02;0.01] | -0.01 | 0.01 | [-0.02;0.01] | -0.01 | -0.66(46) | 0.52 | [-0.02;0.01] |
| Sex (male) | -0.34 | 0.21 | [-0.77;0.06] | -0.35 | 0.22 | [-0.76;0.10] | -0.35 | 0.22 | [-0.77;0.08] | -0.35 | -1.61(46) | 0.11 | [-0.78;-0.09] |
| PSI-Q | -0.07 | 0.07 | [-0.21;0.06] | -0.07 | 0.07 | [-0.22;0.06] | -0.07 | 0.07 | [-0.21;0.07] | -0.07 | -1.08(46) | 0.29 | [-0.21;0.06] |
| MMQ Ability | 0.01 | 0.01 | [-0.02;0.03] | 0.00 | 0.01 | [-0.02;0.03] | 0.00 | 0.01 | [-0.02;0.03] | 0.00 | 0.46(46) | 0.65 | [-0.02;0.03] |
| MMQ Satisfaction | 0.00 | 0.02 | [-0.04;0.04] | 0.00 | 0.02 | [-0.04;0.04] | 0.00 | 0.02 | [-0.04;0.04] | 0.00 | 0.12(46) | 0.91 | [-0.04;0.04] |
| CFMT+ | 0.02 | 0.01 | [0.00;0.05] | 0.02 | 0.01 | [0.00;0.05] | 0.02 | 0.01 | [0.00;0.05] | 0.02 | 2.21(46) | 0.03 | [0.00;0.05] |
| fCFMT+ | 0.03 | 0.01 | [0.00;0.05] | 0.02 | 0.01 | [0.00;0.05] | 0.02 | 0.01 | [0.00;0.05] | 0.03 | 2.16(46) | 0.04 | [0.00;0.05] |
| Age | 0.00 | 0.01 | [-0.02;0.03] | 0.00 | 0.01 | [-0.02;0.03] | 0.00 | 0.01 | [-0.02;0.03] | 0.01 | 0.32(46) | 0.75 | [-0.02;0.03] |
| Dynamic Touc*STQ | 0.00 | 0.00 | [-0.01;0.01] | 0.00 | 0.00 | [-0.01;0.01] | 0.00 | 0.00 | [-0.01;0.01] | 0.00 | -0.30(106) | 0.77 | [-0.01;0.01] |
| Static Touch*STQ | 0.00 | 0.00 | [-0.01;0.01] | 0.00 | 0.00 | [-0.01;0.01] | 0.00 | 0.00 | [-0.01;0.01] | 0.00 | 0.59(106) | 0.55 | [-0.01;0.01] |

Table 25. Model fits for stepwise approach.

|  | df | AIC | BIC | Likelihood ratio |
| --- | --- | --- | --- | --- |
| Empty model + covariates + condition + random slope | 17 | 279.60 | 331.34 | / |
| Empty model + covariates + condition + interaction + random slope | 20 | 310.07 | 370.55 | 24.47, p < 0.001 |

Table 26. Bayes factors for d' moderated by attitudes towards social touch (static versus dynamic versus no touch).

|  | Prior: small effect | Prior: medium effect | Prior: large effect |
| --- | --- | --- | --- |
| BF | 208011.35 | 221460.37 | 183864.94 |

## Moderation by STQ: Confidence Ratings

### Touch versus no touch

Full model

Table 27. Full models for confidence ratings moderated by attitudes towards social touch (touch versus no touch) without random slope.

|  | Bayesian (Prior: small effect) | | | Bayesian (Prior: medium effect) | | | Bayesian (Prior: large effect) | | | Frequentist | | | |
| --- | --- | --- | --- | --- | --- | --- | --- | --- | --- | --- | --- | --- | --- |
|  | Beta | SE | CI | Beta | SE | CI | Beta | SE | CI | Beta | T(df) | p | CI |
| Intercept | 8.33 | 0.17 | [7.99;8.66] | 8.33 | 0.17 | [7.98;8.67] | 8.33 | 0.18 | [7.98;8.67] | 8.33 | 49.33(53) | <.001 | [7.99;8.66] |
| Condition (Touch) | 0.03 | 0.05 | [-0.08;0.13] | 0.03 | 0.05 | [-0.07;0.13] | 0.03 | 0.05 | [-0.08;0.13] | 0.03 | 0.56(53) | 0.58 | [-0.07;0.13] |
| STQ | -0.01 | 0.01 | [-0.03;0.02] | -0.01 | 0.01 | [-0.04;0.02] | -0.01 | 0.01 | [-0.03;0.02] | -0.01 | -0.62(46) | 0.54 | [-0.03;0.02] |
| Sex (male) | 0.15 | 0.33 | [-0.49;0.81] | 0.15 | 0.34 | [-0.49;0.83] | 0.14 | 0.33 | [-0.52;0.80] | 0.15 | 0.45(46) | 0.66 | [-0.51;0.81] |
| PSI-Q | 0.18 | 0.10 | [-0.03;0.39] | 0.18 | 0.10 | [-0.02;0.38] | 0.18 | 0.10 | [-0.03;0.38] | 0.18 | 1.76(46) | 0.09 | [-0.03, 0.39] |
| MMQ Ability | 0.00 | 0.02 | [-0.03;0.03] | 0.00 | 0.02 | [-0.03;0.03] | 0.00 | 0.02 | [-0.03;0.03] | 0.00 | -0.06(46) | 0.95 | [-0.03;0.04] |
| MMQ Satisfaction | 0.01 | 0.03 | [-0.05;0.07] | 0.01 | 0.03 | [-0.04;0.07] | 0.01 | 0.03 | [-0.04;0.07] | 0.01 | 0.43(46) | 0.67 | [-0.05;0.07] |
| CFMT+ | 0.02 | 0.02 | [-0.01;0.06] | 0.02 | 0.02 | [-0.01;0.06] | 0.03 | 0.02 | [-0.01;0.06] | 0.02 | 1.47(46) | 0.15 | [-0.01;0.06] |
| fCFMT+ | 0.02 | 0.02 | [-0.01;0.06] | 0.02 | 0.02 | [-0.02;0.06] | 0.02 | 0.02 | [-0.01;0.06] | 0.02 | 1.22(46) | 0.23 | [-0.01;0.06] |
| Age | 0.01 | 0.02 | [-0.04;0.05] | 0.00 | 0.02 | [-0.04;0.05] | 0.00 | 0.02 | [-0.04;0.05] | 0.00 | 0.23(46) | 0.82 | [-0.04;0.05] |
| Touch*STQ | 0.00 | 0.00 | [-0.01;0.01] | 0.00 | 0.00 | [-0.01;0.01] | 0.00 | 0.00 | [-0.01;0.01] | 0.00 | 0.06(53) | 0.95 | [-0.01;0.01] |

Table 28. Model fits for stepwise approach.

|  | df | AIC | BIC | Likelihood Ratio |
| --- | --- | --- | --- | --- |
| Empty model + covariates + condition + random slope | 13 | 262.76 | 296.76 | 1.25, p = .54 |
| Empty model + covariates + condition + random slope | 15 | 282.41 | 321.34 | 15.65, p < .001 |

Table 29. Bayes factors for confidence ratings moderated by attitudes towards social touch (touch versus no touch).

|  | Prior: small effect | Prior: medium effect | Prior: large effect |
| --- | --- | --- | --- |
| BF | 1910.44 | 2183.59 | 2100.99 |

### Static versus dynamic versus no touch

Table 30. Full models for confidence ratings moderated by attitudes towards social touch (static versus dynamic versus no touch) without random slope.

|  | Bayesian (Prior: small effect) | | | Bayesian (Prior: medium effect) | | | Bayesian (Prior: large effect) | | | Frequentist | | | |
| --- | --- | --- | --- | --- | --- | --- | --- | --- | --- | --- | --- | --- | --- |
|  | Beta | SE | CI | Beta | SE | CI | Beta | SE | CI | Beta | T(df) | p | CI |
| Intercept | 8.32 | 0.17 | [7.99;8.65] | 8.31 | 0.17 | [7.99;8.65] | 8.31 | 0.17 | [7.99;8.65] | 8.32 | 48.95(106) | <0.001 | [7.98;8.65] |
| Condition (Dynamic Touch) | 0.04 | 0.07 | [-0.09;0.17] | 0.04 | 0.07 | [-0.10;0.17] | 0.04 | 0.07 | [-0.09;0.17] | 0.04 | 0.54(106) | 0.59 | [-0.09;0.17] |
| Condition (Static Touch) | 0.02 | 0.07 | [-0.11;0.16] | 0.02 | 0.07 | [-0.11;0.15] | 0.02 | 0.07 | [-0.11;0.15] | 0.02 | 0.33(106) | 0.74 | [-0.11;0.15] |
| STQ | -0.01 | 0.01 | [-0.03;0.02] | -0.01 | 0.01 | [-0.03;0.02] | -0.01 | 0.01 | [-0.03;0.02] | -0.01 | -0.58(46) | 0.56 | [-0.03;0.02] |
| Sex (male) | 0.15 | 0.32 | [-0.50;0.78] | 0.18 | 0.33 | [-0.49;0.82] | 0.18 | 0.33 | [-0.45;0.83] | 0.17 | 0.52(46) | 0.60 | [-0.49;0.83] |
| PSI-Q | 0.17 | 0.10 | [-0.03;0.36] | 0.17 | 0.10 | [-0.02;0.38] | 0.17 | 0.11 | [-0.04;0.38] | 0.17 | 1.67(46) | 0.10 | [-0.04;0.38] |
| MMQ Ability | 0.00 | 0.02 | [-0.03;0.03] | 0.00 | 0.02 | [-0.03;0.03] | 0.00 | 0.02 | [-0.03;0.03] | 0.00 | 0.05(46) | 0.96 | [-0.03,0.03] |
| MMQ Satisfaction | 0.01 | 0.03 | [-0.04;0.07] | 0.01 | 0.03 | [-0.04;0.07] | 0.01 | 0.03 | [-0.04;0.07] | 0.01 | 0.48(46) | 0.64 | [-0.05;0.07] |
| CFMT+ | 0.02 | 0.02 | [-0.01;0.06] | 0.02 | 0.02 | [-0.01;0.06] | 0.02 | 0.02 | [-0.01;0.06] | 0.02 | 1.33(46) | 0.19 | [-0.01;0.06] |
| fCFMT+ | 0.02 | 0.02 | [-0.01;0.06] | 0.02 | 0.02 | [-0.01;0.06] | 0.02 | 0.02 | [-0.01;0.06] | 0.02 | 1.39(46) | 0.17 | [-0.01;0.06] |
| Age | 0.01 | 0.02 | [-0.04;0.05] | 0.01 | 0.02 | [-0.04;0.05] | 0.01 | 0.02 | [-0.04;0.05] | 0.01 | 0.28(46) | 0.78 | [-0.04,0.05] |
| Dynamic Touch*STQ | 0.00 | 0.01 | [-0.01;0.01] | 0.00 | 0.01 | [-0.01;0.01] | 0.00 | 0.01 | [-0.01;0.01] | -0.01 | 0.34(106) | 0.74 | [-0.01;0.01] |
| Static Touch*STQ | 0.00 | 0.01 | [-0.01;0.01] | 0.00 | 0.01 | [-0.01;0.01] | 0.00 | 0.01 | [-0.01;0.01] | 0.00 | -0.24(106) | 0.81 | [-0.01;0.01] |

Table 31. Model fits for stepwise approach.

|  | df | AIC | BIC | Likelihood Ratio |
| --- | --- | --- | --- | --- |
| Empty model + covariates + condition + random slope | 17 | 357.27 | 409.00 | / |
| Empty model + covariates + condition + interaction + random slope | 20 | 387.00 | 447.48 | 23.74, p < .001 |

Table 32. Bayes factors for confidence ratings moderated by attitudes towards social touch (static versus dynamic versus no touch)

|  | Prior: small effect | Prior: medium effect | Prior: large effect |
| --- | --- | --- | --- |
| BF | 125487.32 | 111330.40 | 107682.15 |

## Moderation by STQ: Metacognitive Sensitivity

### Touch versus no touch

Table 33. Full models for meta d' moderated by attitudes towards social touch (touch versus no touch) without random slope.

|  | Bayesian (Prior: small effect) | | | Bayesian (Prior: medium effect) | | | Bayesian (Prior: large effect) | | | Frequentist | | | |
| --- | --- | --- | --- | --- | --- | --- | --- | --- | --- | --- | --- | --- | --- |
|  | Beta | SE | CI | Beta | SE | CI | Beta | SE | CI | Beta | T(df) | p | CI |
| Intercept | 2.25 | 0.15 | [1.95;2.55] | 2.24 | 0.15 | [1.94;2.53] | 2.25 | 0.16 | [1.93;2.56] | 2.25 | 14.91(53) | <.001 | [1.95;2.55] |
| Condition (Touch) | -0.07 | 0.13 | [-0.32;0.17] | -0.07 | 0.13 | [-0.32;0.18] | -0.07 | 0.13 | [-0.33;0.18] | -0.07 | -0.55(53) | -0.58 | [-0.32;0.18] |
| STQ | 0.00 | 0.01 | [-0.02;0.02] | 0.00 | 0.01 | [-0.02;0.02] | 0.00 | 0.01 | [-0.02;0.02] | 0.00 | -0.02(46) | 0.98 | [-0.02;0.02] |
| Sex (male) | -0.41 | 0.27 | [-0.93;0.12] | -0.40 | 0.28 | [-0.93;0.14] | -0.42 | 0.28 | [-0.98;0.12] | -0.42 | -1.54(46) | 0.13 | [-0.96;0.13] |
| PSI-Q | -0.08 | 0.09 | [-0.25;0.09] | -0.08 | 0.08 | [-0.25;0.08] | -0.08 | 0.09 | [-0.25;0.09] | -0.08 | -0.98(46) | 0.33 | [-0.25;0.09] |
| MMQ Ability | 0.02 | 0.01 | [-0.01;0.04] | 0.01 | 0.01 | [-0.01;0.04] | 0.02 | 0.01 | [-0.01;0.04] | 0.01 | 1.12(46) | 0.26 | [-0.01;0.04] |
| MMQ Satisfaction | 0.01 | 0.03 | [-0.04;0.06] | 0.01 | 0.02 | [-0.04;0.06] | 0.01 | 0.03 | [-0.04;0.06] | 0.01 | 0.42(46) | 0.67 | [-0.04;0.06] |
| CFMT+ | 0.02 | 0.01 | [-0.01;0.04] | 0.02 | 0.01 | [-0.01;0.04] | 0.02 | 0.01 | [-0.01;0.04] | 0.02 | 1.16(46) | 0.25 | [-0.01;0.04] |
| fCFMT+ | 0.04 | 0.01 | [0.01;0.06] | 0.04 | 0.02 | [0.01;0.07] | 0.04 | 0.01 | [0.01;0.06] | 0.04 | 2.44(46) | 0.02 | [0.01;0.07] |
| Age | 0.02 | 0.02 | [-0.01;0.06] | 0.02 | 0.02 | [-0.01;0.05] | 0.02 | 0.02 | [-0.02;0.06] | 0.02 | 1.25(46) | 0.22 | [-0.01;0.06] |
| Touch*STQ | -0.01 | 0.01 | [-0.03;0.01] | -0.01 | 0.01 | [-0.04;0.01] | -0.01 | 0.01 | [-0.04;0.01] | -0.01 | -1.23(53) | 0.23 | [-0.03;0.01] |

Table 34. Model fits for stepwise approach.

|  | df | AIC | BIC | Likelihood Ratio |
| --- | --- | --- | --- | --- |
| Empty model + covariates + condition + random slope | 13 | 342.57 | 376.58 | / |
| Empty model + covariates + condition + interaction + random slope | 15 | 358.70 | 397.63 | 12.13, p = 0.002 |

Table 35. Bayes factors for meta d' moderated by attitudes towards social touch (touch versus no touch).

|  | Prior: small effect | Prior: medium effect | Prior: large effect |
| --- | --- | --- | --- |
| BF | 544.32 | 569.62 | 524.68 |

### Static versus dynamic versus no touch

Table 36. Full model for meta d' moderated by attitudes towards social touch (static versus affective versus no touch) without random slope.

|  | Bayesian (Prior: small effect) | | | Bayesian (Prior: medium effect) | | | Bayesian (Prior: large effect) | | | Frequentist | | | |
| --- | --- | --- | --- | --- | --- | --- | --- | --- | --- | --- | --- | --- | --- |
|  | Beta | SE | CI | Beta | SE | CI | Beta | SE | CI | Beta | T(df) | p | CI |
| Intercept | 2.24 | 0.16 | [1.93;2.55] | 2.25 | 0.15 | [1.94;2.55] | 2.25 | 0.16 | [1.93;2.58] | 2.25 | 14.40(106) | <.001 | [1.94; 2.56] |
| Condition (Dynamic Touch) | 0.00 | 0.11 | [-0.22;0.23] | 0.00 | 0.11 | [-0.22;0.22] | 0.00 | 0.12 | [-0.22;0.22] | 0.00 | 0.03(106) | 0.98 | [-0.22;0.23] |
| Condition (Static Touch) | -0.07 | 0.11 | [-0.29;0.15] | -0.06 | 0.11 | [-0.29;0.16] | -0.06 | 0.12 | [-0.30;0.16] | -0.06 | -0.57(106) | 0.57 | [-0.29;0.16] |
| STQ | 0.00 | 0.01 | [-0.03;0.02] | 0.00 | 0.01 | [-0.03;0.02] | 0.00 | 0.01 | [-0.03;0.02] | 0.00 | -0.08(46) | 0.93 | [-0.03;0.02] |
| Sex (male) | -0.42 | 0.29 | [-0.97;0.16] | -0.42 | 0.28 | [-0.97;0.13] | -0.41 | 0.28 | [-0.97;0.13] | -0.42 | -1.50(46) | 0.14 | [-0.98;0.14] |
| PSI-Q | -0.08 | 0.09 | [-0.26;0.09] | -0.08 | 0.09 | [-0.26;0.09] | -0.08 | 0.09 | [-0.25;0.10] | -0.08 | -0.89(46) | 0.38 | [-0.25;0.10] |
| MMQ Ability | 0.01 | 0.01 | [-0.01;0.04] | 0.01 | 0.01 | [-0.02;0.04] | 0.01 | 0.01 | [-0.01;0.04] | 0.01 | 0.94(46) | 0.35 | [-0.01;0.04] |
| MMQ Satisfaction | 0.01 | 0.03 | [-0.04;0.07] | 0.01 | 0.03 | [-0.04;0.06] | 0.01 | 0.03 | [-0.04;0.06] | 0.01 | 0.49(46) | 0.63 | [-0.04;0.06] |
| CFMT+ | 0.02 | 0.01 | [-0.01;0.04] | 0.02 | 0.01 | [-0.01;0.05] | 0.02 | 0.01 | [-0.01;0.05] | 0.02 | 1.14 (46) | 0.26 | [-0.01;0.05] |
| fCFMT+ | 0.04 | 0.01 | [0.01;0.07] | 0.04 | 0.02 | [0.01;0.07] | 0.04 | 0.02 | [0.00;0.07] | 0.04 | 2.42(46) | 0.02 | [0.01;0.07] |
| Age | 0.02 | 0.02 | [-0.01;0.06] | 0.02 | 0.02 | [-0.01;0.06] | 0.02 | 0.02 | [-0.01;0.06] | 0.02 | 1.31(46) | 0.20 | [-0.01;0.06] |
| Dynamic Touch*STQ | -0.02 | 0.01 | [-0.04;0.00] | -0.02 | 0.01 | [-0.04;0.00] | -0.02 | 0.01 | [-0.04;0.00] | -0.02 | -1.72(106) | 0.15 | [-0.04;0.01] |
| Static Touch*STQ | 0.00 | 0.01 | [-0.01;0.02] | 0.00 | 0.01 | [-0.02;0.02] | 0.00 | 0.01 | [-0.02;0.02] | 0.00 | 0.41(106) | 0.68 | [-0.02;0.02] |

Table 37. Model fits for stepwise approach.

|  | df | AIC | BIC | Likelihood Ratio |
| --- | --- | --- | --- | --- |
| Empty model + covariates + condition + random slope | 17 | 460.18 | 511.92 | / |
| Empty model + covariates + condition + interaction + random slope | 20 | 482.69 | 543.17 | 16.51, p < .001 |

Table 38. Bayes factors for meta d' moderated by attitudes towards social touch (static versus dynamic versus no touch).

|  | Prior: small effect | Prior: medium effect | Prior: large effect |
| --- | --- | --- | --- |
| BF | 4861.69 | 4706.54 | 4566.10 |

## Attractiveness Ratings

### Touch versus no touch

Table 39. Full models for difference in attractiveness ratings (touch versus no touch) without random slope.

|  | Bayesian (Prior: small effect) | | | Bayesian (Prior: medium effect) | | | Bayesian (Prior: large effect) | | | Frequentist | | | |
| --- | --- | --- | --- | --- | --- | --- | --- | --- | --- | --- | --- | --- | --- |
|  | Beta | SE | CI | Beta | SE | CI | Beta | SE | CI | Beta | T(df) | p | CI |
| Intercept | -0.07 | 0.06 | [-0.19;0.05] | -0.07 | 0.06 | [-0.19;0.06] | -0.07 | 0.06 | [-0.19;0.06] | -0.07 | -1.07(54) | 0.29 | [-0.19;0.06] |
| Condition (Touch) | 0.01 | 0.07 | [-0.13;0.15] | 0.01 | 0.07 | [-0.13;0.15] | 0.01 | 0.07 | [-0.12;0.15] | 0.01 | 0.17(54) | 0.87 | [-0.12;0.15] |
| Sex (male) | 0.01 | 0.10 | [-0.18;0.21] | 0.01 | 0.10 | [-0.19;0.22] | 0.00 | 0.10 | [-0.19;0.22] | 0.01 | 0.12(47) | 0.91 | [-0.20;0.22] |
| PSI-Q | 0.00 | 0.03 | [-0.07;0.06] | 0.00 | 0.03 | [-0.07;0.06] | 0.00 | 0.03 | [-0.07;0.07] | 0.00 | -0.03(47) | 0.98 | [-0.07;0.06] |
| MMQ Ability | 0.00 | 0.00 | [-0.01;0.01] | 0.00 | 0.01 | [-0.01;0.01] | 0.00 | 0.01 | [-0.01;0.01] | 0.00 | 0.57(47) | 0.57 | [-0.01;0.01] |
| MMQ Satisfaction | 0.00 | 0.01 | [-0.01;0.02] | 0.00 | 0.01 | [-0.01;0.02] | 0.00 | 0.01 | [-0.02;0.02] | 0.00 | 0.39(47) | 0.70 | [-0.02;0.02] |
| CFMT+ | 0.00 | 0.01 | [-0.01;0.02] | 0.00 | 0.01 | [-0.01;0.02] | 0.00 | 0.01 | [-0.01;0.02] | 0.00 | 0.90(47) | 0.37 | [-0.01;0.02] |
| fCFMT+ | -0.01 | 0.01 | [-0.02;0.01] | -0.01 | 0.01 | [-0.02;0.01] | -0.01 | 0.01 | [-0.02;0.01] | -0.01 | -0.99(47) | 0.33 | [-0.02;0.00] |
| Age | 0.00 | 0.01 | [-0.01;0.01] | 0.00 | 0.01 | [-0.01;0.01] | 0.00 | 0.01 | [-0.01;0.01] | 0.00 | -0.03(47) | 0.98 | [-0.01;0.01] |

Table 40. Model fits for stepwise approach.

|  | df | AIC | BIC | Likelihood ratio |
| --- | --- | --- | --- | --- |
| Empty model + covariates | 10 | 177.65 | 203.90 | / |
| Empty model + covariates + condition | 11 | 183.20 | 211.96 | 3.55, p = 0.06 |
| Empty model + covariates + condition + random slope | 13 | 187.18 | 221.18 | 0.01, p = 0.99 |

Table 41. Bayes factors for differences attractiveness ratings (touch versus no touch).

|  | Prior: small effect | Prior: medium effect | Prior: large effect |
| --- | --- | --- | --- |
| BF | 5.28 | 6.29 | 6.10 |

### Static versus dynamic versus no touch

Table 42. Full models for difference in attractiveness ratings (static versus dynamic versus no touch) without random slope.

|  | Bayesian (Prior: small effect) | | | Bayesian (Prior: medium effect) | | | Bayesian (Prior: large effect) | | | Frequentist | | | |
| --- | --- | --- | --- | --- | --- | --- | --- | --- | --- | --- | --- | --- | --- |
|  | Beta | SE | CI | Beta | SE | CI | Beta | SE | CI | Beta | T(df) | p | CI |
| Intercept | -0.08 | 0.07 | [-0.21;0.05] | -0.08 | 0.07 | [-0.21;0.06] | -0.08 | 0.07 | [-0.22;0.05] | -0.08 | -1.18(108) | 0.24 | [-0.21;0.05] |
| Condition (Dynamic Touch) | 0.03 | 0.07 | [-0.10;0.16] | 0.03 | 0.07 | [-0.11;0.16] | 0.03 | 0.07 | [-0.11;0.17] | 0.03 | 0.39(108) | 0.70 | [-0.11;0.16] |
| Condition (Static Touch) | 0.00 | 0.07 | [-0.14;0.14] | 0.00 | 0.07 | [-0.13;0.13] | 0.00 | 0.07 | [-0.14;0.13] | 0.00 | -0.06(108) | 0.95 | [-0.14;0.13] |
| Sex (male) | 0.06 | 0.10 | [-0.15;0.26] | 0.05 | 0.11 | [-0.16;0.26] | 0.05 | 0.11 | [-0.16;0.27] | 0.05 | 0.51(47) | 0.61 | [-0.16;0.27] |
| PSI-Q | 0.00 | 0.03 | [-0.06;0.07] | 0.01 | 0.03 | [-0.06;0.07] | 0.00 | 0.03 | [-0.06;0.07] | 0.00 | 0.10(47) | 0.92 | [-0.06;0.07] |
| MMQ Ability | 0.00 | 0.01 | [-0.01;0.01] | 0.00 | 0.01 | [-0.01;0.01] | 0.00 | 0.01 | [-0.01;0.01] | 0.00 | 0.40(47) | 0.69 | [-0.01;0.01] |
| MMQ Satisfaction | 0.00 | 0.01 | [-0.02;0.02] | 0.00 | 0.01 | [-0.02;0.02] | 0.00 | 0.01 | [-0.02;0.02] | 0.00 | 0.03(47) | 0.98 | [-0.02;0.02] |
| CFMT+ | 0.01 | 0.01 | [-0.01;0.01] | 0.01 | 0.01 | [-0.01;0.02] | 0.01 | 0.01 | [-0.01;0.02] | 0.00 | 0.97(47) | 0.34 | [-0.01;0.02] |
| fCFMT+ | 0.00 | 0.01 | [-0.02;0.01] | 0.00 | 0.01 | [-0.02;0.01] | 0.00 | 0.01 | [-0.02;0.01] | 0.00 | -0.81(47) | 0.42 | [-0.01;0.01] |
| Age | 0.00 | 0.01 | [-0.01;0.01] | 0.00 | 0.01 | [-0.01;0.01] | 0.00 | 0.01 | [-0.01;0.01] | 0.00 | 0.06(47) | 0.95 | [-0.01;0.01] |

Table 43. Model fits for stepwise approach.

|  | df | AIC | BIC | Likelihood ratio |
| --- | --- | --- | --- | --- |
| Empty model + covariates | 10 | 245.74 | 276.31 | / |
| Empty model + covariates + condition | 12 | 256.90 | 293.43 | 7.16, p =0.03 |
| Empty model + covariates + condition + random slope | 17 | 254.24 | 305.98 | 12.67, p =0.03 |

Table 44. Bayes factors for differences in attractiveness ratings (static versus dynamic versus no touch).

|  | Prior: small effect | Prior: medium effect | Prior: large effect |
| --- | --- | --- | --- |
| BF | 35.66 | 34.32 | 35.29 |

## Trustworthiness Ratings

### Touch versus no touch

Table 45. Full models for differences in trustworthiness ratings (touch verus no touch) with random slope.

|  | Bayesian (Prior: small effect) | | | Bayesian (Prior: medium effect) | | | Bayesian (Prior: large effect) | | | Frequentist | | | |
| --- | --- | --- | --- | --- | --- | --- | --- | --- | --- | --- | --- | --- | --- |
|  | Beta | SE | CI | Beta | SE | CI | Beta | SE | CI | Beta | T(df) | p | CI |
| Intercept | -0.24 | 0.06 | [-0.36;-0.12] | -0.23 | 0.06 | [-0.35;-0.12] | -0.23 | 0.06 | [-0.35;-0.12] | -0.23 | -4.15(54) | <.001 | [-0.34,-0.12] |
| Condition (Touch) | 0.02 | 0.09 | [-0.17;0.20] | 0.02 | 0.09 | [-0.16;0.20] | 0.01 | 0.09 | [-0.16;0.19] | 0.02 | 0.18(54) | 0.86 | [-0.17;0.20] |
| Sex (male) | 0.01 | 0.10 | [-0.18; 0.21] | 0.01 | 0.11 | [-0.20;0.23] | 0.01 | 0.10 | [-0.19;0.21] | 0.00 | 0.03(47) | 0.98 | [-0.20;0.20] |
| PSI-Q | -0.02 | 0.03 | [-0.08; 0.05] | -0.02 | 0.03 | [-0.08;0.05] | -0.02 | 0.03 | [-0.08;0.05] | -0.02 | -0.66(47) | 0.52 | [-0.08;0.04] |
| MMQ Ability | 0.00 | 0.01 | [-0.01; 0.01] | 0.00 | 0.01 | [-0.01;0.01] | 0.00 | 0.01 | [-0.01;0.01] | 0.00 | 0.54(47) | 0.59 | [-0.01; 0.01] |
| MMQ Satisfaction | -0.01 | 0.01 | [-0.03; 0.01] | -0.01 | 0.01 | [-0.03;0.01] | -0.01 | 0.01 | [-0.03;0.01] | -0.01 | -0.97(47) | 0.34 | [-0.03; 0.01] |
| CFMT+ | 0.00 | 0.01 | [-0.01; 0.01] | 0.00 | 0.01 | [-0.01;0.01] | 0.00 | 0.01 | [-0.01;0.01] | 0.00 | -0.23(47) | 0.82 | [-0.01; 0.01] |
| fCFMT+ | -0.01 | 0.01 | [-0.02; 0.01] | -0.01 | 0.01 | [-0.02;0.01] | -0.01 | 0.01 | [-0.02;0.01] | 0.00 | -0.91(47) | 0.37 | [-0.02;0.01] |
| Age | -0.01 | 0.01 | [-0.02; 0.01] | -0.01 | 0.01 | [-0.02;0.01] | -0.01 | 0.01 | [-0.02;0.01] | -0.01 | -0.78(47) | 0.44 | [-0.02; 0.01] |

Table 46. Model fits for stepwise approach.

|  | df | AIC | BIC | Likelihood Ratio |
| --- | --- | --- | --- | --- |
| Empty model + covariates | 10 | 218.76 | 245.01 | / |
| Empty model + covariates + condition | 11 | 223.70 | 252.47 | 2.94, p =0.09 |
| Empty model + covariates + condition + random slope | 13 | 216.65 | 250.65 | 11.05, p = 0.004 |

Table 47. Bayes factors for differences in trustworthiness ratings (touch versus no touch).

|  | Prior: small effect | Prior: medium effect | Prior: large effect |
| --- | --- | --- | --- |
| BF | 0.11 | 0.20 | 0.10 |

### Static versus dynamic versus no touch

Table 48. Full models for differences in trustworthiness ratings (static versus dynamic versus no touch) with random slope.

|  | Bayesian (Prior: small effect) | | | Bayesian (Prior: medium effect) | | | Bayesian (Prior: large effect) | | | Frequentist | | | |
| --- | --- | --- | --- | --- | --- | --- | --- | --- | --- | --- | --- | --- | --- |
|  | Beta | SE | CI | Beta | SE | CI | Beta | SE | CI | Beta | T(df) | p | CI |
| Intercept | -0.24 | 0.06 | [-0.35;-0.12] | -0.24 | 0.06 | [-0.36;-0.12] | -0.23 | 0.06 | [-0.35;-0.12] | -0.23 | -4.12(108) | < .001 | [-0.34;-0.12] |
| Condition (Dynamic Touch) | -0.08 | 0.10 | [-0.27; 0.12] | -0.09 | 0.10 | [-0.29; 0.11] | -0.09 | 0.10 | [-0.29; 0.11] | -0.09 | -0.89(108) | 0.38 | [-0.28; 0.11] |
| Condition (Static Touch) | 0.12 | 0.11 | [-0.09;0.35] | 0.12 | 0.11 | [-0.10;0.34] | 0.12 | 0.11 | [-0.11;0.33] | 0.12 | 1.06(47) | 0.98 | [-0.10;0.34] |
| Sex (male) | 0.02 | 0.11 | [-0.18;0.23] | 0.02 | 0.11 | [-0.20;0.23] | 0.01 | 0.10 | [-0.19;0.22] | 0.00 | -0.03(47) | 0.98 | [-0.20;0.20] |
| PSI-Q | -0.02 | 0.03 | [-0.08;0.05] | -0.02 | 0.03 | [-0.09;0.05] | -0.02 | 0.03 | [-0.08;0.05] | -0.02 | -0.61(47) | 0.55 | [-0.08;0.04] |
| MMQ Ability | 0.00 | -0.01 | [-0.01;0.01] | 0.00 | -0.01 | [-0.01;0.01] | 0.00 | -0.01 | [-0.01;0.01] | 0.00 | -1.04(47) | 0.71 | [-0.01;0.01] |
| MMQ Satisfaction | -0.01 | -0.01 | [-0.03;0.01] | -0.01 | -0.01 | [-0.03;0.01] | -0.01 | -0.01 | [-0.03;0.01] | 0.00 | -1.04(47) | 0.31 | [-0.03;0.01] |
| CFMT+ | -0.00 | -0.01 | [-0.01;0.01] | -0.00 | -0.01 | [-0.01;0.01] | -0.00 | -0.01 | [-0.01;0.01] | 0.00 | -0.04(47) | 0.97 | [-0.01;0.01] |
| fCFMT+ | -0.01 | -0.01 | [-0.02;0.01] | -0.01 | -0.01 | [-0.02;0.01] | -0.01 | -0.01 | [-0.02;0.01] | 0.01 | -1.12(47) | 0.27 | [-0.02;0.01] |
| Age | -0.01 | -0.01 | [-0.02;0.01] | -0.01 | -0.01 | [-0.02;0.01] | -0.01 | -0.01 | [-0.02;0.01] | -0.01 | -0.79(47) | 0.43 | [-0.02;0.01] |

Table 49. Model fits for stepwise approach.

|  | df | AIC | BIC | Likelihood Ratio |
| --- | --- | --- | --- | --- |
| Empty model + covariates | 10 | 365.10 | 395.66 | / |
| Empty model + covariates + condition | 12 | 370.91 | 407.43 | 1.81, p = 0.40 |
| Empty model + covariates + condition + random slope | 17 | 352.99 | 404.73 | 27.92, p < 0001 |

Table 50. Bayes factors for differences in trustworthiness ratings (static versus dynamic versus no touch).

|  | Prior: small effect | Prior: medium effect | Prior: large effect |
| --- | --- | --- | --- |
| BF | 0.001 | 0.002 | 0.004 |

## Moderation by STQ: Attractiveness Ratings

### Touch versus no touch

Table 51. Full models for difference in attractiveness ratings moderated by attitudes towards social touch (touch versus no touch) without random slope.

|  | Bayesian (Prior: small effect) | | | Bayesian (Prior: medium effect) | | | Bayesian (Prior: large effect) | | | Frequentist | | | |
| --- | --- | --- | --- | --- | --- | --- | --- | --- | --- | --- | --- | --- | --- |
|  | Beta | SE | CI | Beta | SE | CI | Beta | SE | CI | Beta | T(df) | p | CI |
| Intercept | -0.07 | 0.06 | [-0.20;0.05] | -0.07 | 0.06 | [-0.20;0.06] | -0.07 | 0.06 | [-0.20;0.05] | -0.07 | -1.10(53) | 0.28 | [-0.20;0.06] |
| Condition (Touch) | 0.01 | 0.07 | [-0.13;0.15] | 0.01 | 0.07 | [-0.13;0.15] | 0.01 | 0.07 | [-0.13;0.15] | 0.01 | 0.14(53) | 0.89 | [-0.12;0.14] |
| STQ | 0.01 | 0.01 | [0.00;0.02] | 0.01 | 0.01 | [0.00;0.02] | 0.01 | 0.01 | [0.00;0.02] | 0.01 | 1.20(46) | 0.24 | [0.00;0.02] |
| Sex (male) | 0.02 | 0.10 | [-0.18;0.23] | 0.02 | 0.11 | [-0.18;0.24] | 0.02 | 0.11 | [-0.19;0.24] | 0.02 | 0.22(46) | 0.82 | [-0.19;0.23] |
| PSI-Q | 0.00 | 0.03 | [-0.07;0.07] | 0.00 | 0.03 | [-0.06;0.06] | 0.00 | 0.03 | [-0.06;0.07] | 0.00 | 0.03(46) | 0.97 | [-0.07;0.07] |
| MMQ Ability | 0.00 | 0.01 | [-0.01;0.01] | 0.00 | 0.01 | [-0.01;0.01] | 0.00 | 0.01 | [-0.01;0.01] | 0.00 | 0.66(46) | 0.51 | [-0.01;0.01] |
| MMQ Satisfaction | 0.00 | 0.01 | [-0.01;0.02] | 0.00 | 0.01 | [-0.02;0.02] | 0.00 | 0.01 | [-0.02;0.02] | 0.00 | 0.37(46) | 0.72 | [-0.02;0.02] |
| CFMT+ | 0.00 | 0.01 | [-0.01;0.02] | 0.00 | 0.01 | [-0.01;0.02] | 0.00 | 0.01 | [-0.01;0.02] | 0.00 | 0.83(46) | 0.41 | [-0.01;0.02] |
| fCFMT+ | -0.01 | 0.01 | [-0.02;0.01] | -0.01 | 0.01 | [-0.02;0.01] | -0.01 | 0.01 | [-0.02;0.01] | 0.01 | -0.94(46) | 0.35 | [-0.02;0.01] |
| Age | 0.00 | 0.01 | [-0.01;0.01] | 0.00 | 0.01 | [-0.01;0.01] | 0.00 | 0.01 | [-0.01;0.01] | 0.00 | -0.01(46) | 0.99 | [-0.01;0.01] |
| Touch*STQ | -0.01 | 0.01 | [-0.02;0.01] | -0.01 | 0.01 | [-0.02;0.00] | -0.01 | 0.01 | [-0.02;0.01] | -0.01 | -1.23(53) | 0.23 | [-0.02;0.00] |

Table 52. Model fits for stepwise approach.

|  | df | AIC | BIC | Likelihood ratio |
| --- | --- | --- | --- | --- |
| Empty model + covariates + condition + random slope | 13 | 187.18 | 221.18 | 0.01, p = 0.99 |
| Empty model + covariates + condition + interaction + random slope | 15 | 206.93 | 245.86 | 15.75, p < .001 |

Table 53. Bayes factors for difference in attractiveness ratings moderated by attitudes towards social touch (touch versus no touch).

|  | Prior: small effect | Prior: medium effect | Prior: large effect |
| --- | --- | --- | --- |
| BF | 2491.15 | 2482.58 | 2469.81 |

### Static versus dynamic versus no touch

Table 54. Full models for difference in attractiveness ratings moderated by attitudes towards social touch (static versus dynamic versus no touch) without random slope.

|  | Bayesian (Prior: small effect) | | | Bayesian (Prior: medium effect) | | | Bayesian (Prior: large effect) | | | Frequentist | | | |
| --- | --- | --- | --- | --- | --- | --- | --- | --- | --- | --- | --- | --- | --- |
|  | Beta | SE | CI | Beta | SE | CI | Beta | SE | CI | Beta | T(df) | p | CI |
| Intercept | -0.08 | 0.07 | [-0.22;0.05] | -0.08 | 0.07 | [-0.22;0.05] | -0.08 | 0.07 | [-0.22;0.06] | -0.08 | -1.19(106) | 0.24 | [-0.21;0.05] |
| Condition (Dynamic Touch) | 0.02 | 0.07 | [-0.11;0.16] | 0.02 | 0.07 | [-0.11;0.16] | 0.03 | 0.07 | [-0.11;0.16] | 0.02 | 0.36(106) | 0.72 | [-0.11;0.16] |
| Condition (Static Touch) | -0.01 | 0.07 | [-0.14;0.13] | -0.01 | 0.07 | [-0.14;0.13] | 0.00 | 0.07 | [-0.14;0.13] | -0.01 | -0.08(106) | 0.94 | [-0.14;0.13] |
| STQ | 0.01 | 0.01 | [0.00;0.02] | 0.01 | 0.01 | [0.00;0.02] | 0.01 | 0.01 | [0.00;0.02] | 0.01 | 1.18(46) | 0.24 | [0.00;0.02] |
| Sex (male) | 0.07 | 0.11 | [-0.15;0.29] | 0.06 | 0.11 | [-0.15;0.28] | 0.06 | 0.11 | [-0.15;0.28] | 0.06 | 0.57(46) | 0.57 | [-0.16;0.28] |
| PSI-Q | 0.01 | 0.03 | [-0.06;0.08] | 0.00 | 0.03 | [-0.06;0.07] | 0.00 | 0.03 | [-0.06;0.07] | 0.00 | 0.14(46) | 0.89 | [-0.06;0.07] |
| MMQ Ability | 0.00 | 0.01 | [-0.01;0.01] | 0.00 | 0.01 | [-0.01;0.01] | 0.00 | 0.01 | [-0.01;0.01] | 0.00 | 0.45(46) | 0.65 | [-0.01;0.01] |
| MMQ Satisfaction | 0.00 | 0.01 | [-0.02;0.02] | 0.00 | 0.01 | [-0.02;0.02] | 0.00 | 0.01 | [-0.02;0.02] | 0.01 | 0.01(46) | 0.99 | [-0.02;0.02] |
| CFMT+ | 0.01 | 0.01 | [-0.01;0.02] | 0.01 | 0.01 | [-0.01;0.02] | 0.01 | 0.01 | [-0.01;0.01] | 0.01 | 0.92(46) | 0.36 | [-0.01;0.02] |
| fCFMT+ | 0.00 | 0.01 | [-0.02;0.01] | 0.00 | 0.01 | [-0.02;0.01] | 0.00 | 0.01 | [-0.02;0.01] | 0.00 | -0.77(46) | 0.45 | [-0.02;0.01] |
| Age | 0.00 | 0.01 | [-0.01;0.01] | 0.00 | 0.01 | [-0.01;0.01] | 0.00 | 0.01 | [-0.01;0.01] | 0.00 | 0.07(46) | 0.95 | [-0.01;0.01] |
| Dynamic Touch*STQ | -0.01 | 0.01 | [-0.02;0.00] | -0.01 | 0.01 | [-0.02;0.00] | 0.01 | 0.01 | [-0.02;0.00] | -0.01 | -1.39(106) | 0.17 | [-0.02;0.00] |
| Static Touch*STQ | -0.01 | 0.01 | [-0.02;0.01] | -0.01 | 0.01 | [-0.02;0.01] | 0.01 | 0.01 | [-0.02;0.01] | -0.01 | -1.03(106) | 0.30 | [-0.02;0.01] |

Table 55. Model fits for stepwise approach.

|  | df | AIC | BIC | Likelihood ratio |
| --- | --- | --- | --- | --- |
| Empty model + covariates + condition + random slope | 17 | 254.24 | 305.98 | / |
| Empty model + covariates + condition + interaction + random slope | 20 | 284.36 | 344.83 | 24.12, p < .001 |

Table 56. Bayes Factors for difference in attractiveness ratings moderated by attitudes towards social touch (static versus dynamic versus no touch).

|  | Prior: small effect | Prior: medium effect | Prior: large effect |
| --- | --- | --- | --- |
| BF | 182629.57 | 147966.27 | 150658.81 |

## Moderation by STQ: Trustworthiness Ratings

### Touch versus no touch

Table 57. Full models for difference in trustworthiness ratings moderated by attitudes towards social touch (touch versus no touch).

|  | Bayesian (Prior: small effect) | | | Bayesian (Prior: medium effect) | | | Bayesian (Prior: large effect) | | | Frequentist | | | |
| --- | --- | --- | --- | --- | --- | --- | --- | --- | --- | --- | --- | --- | --- |
|  | Beta | SE | CI | Beta | SE | CI | Beta | SE | CI | Beta | T(df) | p | CI |
| Intercept | -0.23 | 0.06 | [-0.35;-0.11] | -0.23 | 0.06 | [-0.35;-0.12] | -0.23 | 0.06 | [-0.35;-0.12] | -0.23 | -4.07(53) | <.001 | [-0.34,-0.12] |
| Condition (Touch) | 0.01 | 0.09 | [-0.16;0.20] | 0.02 | 0.09 | [-0.16;0.20] | 0.01 | 0.09 | [-0.17;0.19] | 0.01 | 0.16(53) | 0.88 | [-0.17;0.19] |
| STQ | 0.00 | 0.00 | [-0.01; 0.01] | 0.00 | 0.00 | [-0.01; 0.01] | 0.00 | 0.00 | [-0.01; 0.01] | 0.00 | 0.38(46) | 0.70 | [-0.01; 0.01] |
| Sex (male) | 0.00 | 0.11 | [-0.21; 0.22] | 0.01 | 0.11 | [-0.20;0.22] | 0.01 | 0.11 | [-0.21;0.22] | 0.00 | 0.01(46) | 0.99 | [-0.21;0.20] |
| PSI-Q | -0.02 | 0.03 | [-0.08; 0.05] | -0.02 | 0.03 | [-0.09;0.05] | -0.02 | 0.03 | [-0.09;0.05] | -0.02 | -0.67(46) | 0.51 | [-0.09;0.04] |
| MMQ Ability | 0.00 | 0.01 | [-0.01; 0.01] | 0.00 | 0.01 | [-0.01;0.01] | 0.00 | 0.01 | [-0.01;0.01] | 0.00 | 0.48(46) | 0.63 | [-0.01; 0.01] |
| MMQ Satisfaction | -0.01 | 0.01 | [-0.03; 0.01] | -0.01 | 0.01 | [-0.03;0.01] | -0.01 | 0.01 | [-0.03;0.01] | -0.01 | -0.96(46) | 0.35 | [-0.03; 0.01] |
| CFMT+ | 0.00 | 0.01 | [-0.01; 0.01] | 0.00 | 0.01 | [-0.01;0.01] | 0.00 | 0.01 | [-0.01;0.01] | 0.00 | -0.20(46) | 0.85 | [-0.02; 0.01] |
| fCFMT+ | -0.01 | 0.01 | [-0.02; 0.01] | -0.01 | 0.01 | [-0.02;0.01] | -0.01 | 0.01 | [-0.02;0.01] | -0.01 | -0.92(46) | 0.36 | [-0.02;0.01] |
| Age | -0.01 | 0.01 | [-0.02; 0.01] | -0.01 | 0.01 | [-0.02;0.01] | -0.01 | 0.01 | [-0.02;0.01] | -0.01 | -0.79(46) | 0.44 | [-0.02; 0.01] |
| Touch*STQ | -0.01 | 0.01 | [-0.02; 0.01] | -0.01 | 0.01 | [-0.02; 0.01] | -0.01 | 0.01 | [-0.02; 0.01] | -0.01 | -1.38(53) | 0.17 | [-0.03; 0.00] |

Table 58. Model fits for stepwise approach.

|  | df | AIC | BIC | Likelihood Ratio |
| --- | --- | --- | --- | --- |
| Empty model + covariates + condition + random slope | 13 | 216.65 | 250.65 | / |
| Empty model + covariates + condition + interaction + random slope | 15 | 235.80 | 274.73 | 15.15, p < .001 |

Table 59. Bayes factors for difference in trustworthiness ratings moderated by attitudes towards social touch (touch versus no touch).

|  | Prior: small effect | Prior: medium effect | Prior: large effect |
| --- | --- | --- | --- |
| BF | 2436.29 | 1391.94 | 1980.52 |

### Static versus dynamic versus no touch

Table 60. Full models for difference in trustworthiness ratings moderated by attitudes towards social touch (static versus dynamic versus no touch).

|  | Bayesian (Prior: small effect) | | | Bayesian (Prior: medium effect) | | | Bayesian (Prior: large effect) | | | Frequentist | | | |
| --- | --- | --- | --- | --- | --- | --- | --- | --- | --- | --- | --- | --- | --- |
|  | Beta | SE | CI | Beta | SE | CI | Beta | SE | CI | Beta | T(df) | p | CI |
| Intercept | -0.23 | 0.06 | [-0.35;-0.12] | -0.23 | 0.06 | [-0.35;-0.11] | -0.23 | 0.06 | [-0.35;-0.11] | -0.23 | -4.04(106) | < .001 | [-0.34;-0.12] |
| Condition (Dynamic Touch) | -0.09 | 0.10 | [-0.29; 0.10] | -0.09 | 0.10 | [-0.28; 0.11] | -0.09 | 0.10 | [-0.29; 0.09] | -0.09 | -0.94(106) | 0.32 | [-0.28; 0.10] |
| Condition (Static Touch) | 0.11 | 0.11 | [-0.10;0.34] | 0.12 | 0.11 | [-0.11;0.34] | 0.11 | 0.11 | [-0.10;0.33] | 0.12 | 1.04(46) | 0.30 | [-0.11;0.34] |
| STQ | 0.00 | 0.00 | [-0.01;0.01] | 0.00 | 0.00 | [-0.01;0.01] | 0.00 | 0.00 | [-0.01;0.01] | 0.00 | -0.34(46) | 0.73 | [-0.01;0.01] |
| Sex (male) | 0.01 | 0.11 | [-0.20;0.23] | 0.01 | 0.11 | [-0.20;0.22] | 0.01 | 0.11 | [-0.19;0.23] | -0.01 | -0.06(46) | 0.95 | [-0.21;0.20] |
| PSI-Q | -0.02 | 0.03 | [-0.08;0.05] | -0.02 | 0.03 | [-0.08;0.05] | -0.02 | 0.03 | [-0.08;0.05] | -0.02 | -0.62(46) | 0.54 | [-0.08;0.04] |
| MMQ Ability | 0.00 | 0.01 | [-0.01;0.01] | 0.00 | 0.01 | [-0.01;0.01] | 0.00 | 0.01 | [-0.01;0.01] | 0.00 | -1.02(46) | 0.74 | [-0.01;0.01] |
| MMQ Satisfaction | -0.01 | 0.01 | [-0.03;0.01] | -0.01 | 0.01 | [-0.03;0.01] | -0.01 | 0.01 | [-0.03;0.01] | -0.01 | -1.02(46) | 0.31 | [-0.03;0.01] |
| CFMT+ | 0.00 | 0.01 | [-0.01;0.01] | 0.00 | 0.01 | [-0.01;0.01] | 0.00 | 0.01 | [-0.01;0.01] | 0.00 | -0.02(46) | 0.98 | [-0.01;0.01] |
| fCFMT+ | -0.01 | 0.01 | [-0.02;0.01] | -0.01 | 0.01 | [-0.02;0.01] | -0.01 | 0.01 | [-0.02;0.01] | 0.01 | -1.12(47) | 0.27 | [-0.02;0.01] |
| Age | -0.01 | 0.01 | [-0.02;0.01] | -0.01 | 0.01 | [-0.02;0.01] | -0.01 | 0.01 | [-0.02;0.01] | -0.01 | -0.80(46) | 0.43 | [-0.02;0.01] |
| Dynamic Touch * STQ | -0.01 | 0.01 | [-0.03;0.00] | -0.01 | 0.01 | [-0.03;0.00] | -0.01 | 0.01 | [-0.03;0.00] | -0.01 | -1.68(106) | 0.10 | [-0.03;0.00] |
| Static Touch*STQ | -0.01 | 0.01 | [-0.03;0.01] | -0.01 | 0.01 | [-0.03;0.01] | -0.01 | 0.01 | [-0.03;0.01] | -0.01 | -0.78(106) | 0.44 | [-0.03;0.01] |

Table 61. Model fit for stepwise approach.

|  | df | AIC | BIC | Likelihood Ratio |
| --- | --- | --- | --- | --- |
| Empty model + covariates + condition + random slope | 17 | 352.99 | 404.73 | / |
| Empty model + covariates + condition + interaction + random slope | 20 | 380.86 | 441.33 | 21.86, p <.001 |

Table 62. Bayes factors for difference in trustworthiness ratings moderated by attitudes towards social touch (static versus dynamic versus no touch).

|  | Prior: small effect | Prior: medium effect | Prior: large effect |
| --- | --- | --- | --- |
| BF | 575258.93 | 12241.75 | 59430.85 |

## Moderation by Baseline Rating: Attractiveness Ratings

### Touch versus no touch

Table 63. Full models of differences in attractiveness ratings after touch moderated by baseline rating. Item-level regression to be able to include the baseline rating as moderator.

|  | Bayesian (Prior: small effect) | | | Bayesian (Prior: medium effect) | | | Bayesian (Prior: large effect) | | | Frequentist | | | |
| --- | --- | --- | --- | --- | --- | --- | --- | --- | --- | --- | --- | --- | --- |
|  | Beta | SE | CI | Beta | SE | CI | Beta | SE | CI | Beta | T(df) | p | CI |
| Intercept | 0.73 | 0.09 | [0.55;0.91] | 0.73 | 0.09 | [0.55;0.91] | 0.73 | 0.09 | [0.52;0.88] | 0.73 | -7.93  (5222) | < .001 | [-0.55;0.91] |
| Condition (Touch) | -0.03 | 0.08 | [-0.18;0.14] | 0.03 | 0.08 | [-0.18;0.13] | -0.02 | 0.08 | [-0.17;0.23] | -0.03 | -0.31  (5222) | 0.27 | [-0.28;0.08] |
| Baseline attractiveness | -0.18 | 0.01 | [-0.21;-0.15] | -0.18 | 0.01 | [-0.21;-0.15] | -0.18 | 0.01 | [-0.20;-0.14] | -0.18 | -13.58  (5222) | < .001 | [0.00;0.02] |
| Sex (male) | 0.12 | 0.13 | [-0.13;-0.38] | 0.12 | 0.13 | [-0.12;-0.13] | 0.11 | 0.13 | [-0.18;-0.32] | 0.12 | 0.93(47) | 0.36 | [-0.14;0.37] |
| PSI-Q | 0.02 | 0.04 | [-0.06;0.10] | 0.02 | 0.04 | [-0.02;0.04] | 0.02 | 0.04 | [-0.07;0.09] | 0.02 | 0.50(47) | 0.62 | [-0.06;0.10] |
| MMQ Ability | 0.01 | 0.01 | [-0.01;0.02] | 0.01 | 0.01 | [0.01;0.01] | 0.01 | 0.01 | [0.00;0.02] | 0.01 | 1.16(47) | 0.25 | [-0.01;0.02] |
| MMQ Satisfaction | 0.00 | 0.01 | [-0.02;0.03] | 0.00 | 0.01 | [0.00;0.01] | 0.00 | 0.01 | [-0.02;0.03] | 0.00 | 0.28(47) | 0.78 | [-0.02;0.03] |
| CFMT+ | 0.00 | 0.01 | [-0.01;0.02] | 0.00 | 0.01 | [0.00;0.01] | 0.00 | 0.01 | [-0.01;0.01] | 0.00 | 0.42(47) | 0.68 | [-0.01;0.02] |
| fCFMT+ | 0.00 | 0.01 | [-0.02;0.01] | 0.00 | 0.01 | [0.00;0.01] | 0.00 | 0.01 | [-0.02;0.01] | 0.00 | -0.29(47) | 0.77 | [-0.02;0.01] |
| Age | 0.00 | 0.01 | [-0.02;0.02] | 0.00 | 0.01 | [0.01;0.01] | 0.00 | 0.01 | [-0.02;0.02] | 0.00 | 0.00  (47) | 0.99 | [-0.02;0.02] |
| Touch*Baseline attractiveness | 0.01 | 0.02 | [-0.02;0.04] | 0.00 | 0.02 | [0.01;0.02] | 0.01 | 0.02 | [-0.04;0.03] | -0.01 | 0.61  (5222) | 0.54 | [-0.02;0.04] |

Table 64. Model fits for stepwise approach. Note: improved model fit for model including moderation is trivial, since the baseline rating is part of the difference.

|  | df | AIC | BIC | Likelihood ratio |
| --- | --- | --- | --- | --- |
| Empty model + covariates | 10 | 17825.10 | 17890.80 | / |
| Empty model + covariates + condition | 11 | 17831.74 | 17904.01 | 4.64, p = 0.3 |
| Empty model + covariates + condition + interaction | 13 | 17459.82 | 17545.23 | 375.91,  p < .001 |
| Empty model + covariates + condition + interaction + random slope | Does not converge | | | |

Table 65. Bayes factors for differences in trustworthiness ratings after touch moderated by baseline rating. Note: technically this improvement in model fit for model including moderation is trivial, since the baseline rating is part of the difference.

|  | Prior: small effect | Prior: medium effect | Prior: large effect |
| --- | --- | --- | --- |
| BF | < .01 | < .01 | < .01 |

### Static versus dynamic versus no touch

Table 66. Full models of differences in attractiveness ratings after touch moderated by baseline rating. Item-level regression to be able to include the baseline rating as moderator.

|  | Bayesian (Prior: small effect) | | | Bayesian (Prior: medium effect) | | | Bayesian (Prior: large effect) | | | Frequentist | | | |
| --- | --- | --- | --- | --- | --- | --- | --- | --- | --- | --- | --- | --- | --- |
|  | Beta | SE | CI | Beta | SE | CI | Beta | SE | CI | Beta | T(df) | p | CI |
| Intercept | 0.73 | 0.09 | [0.22;0.90] | 0.73 | 0.09 | [0.55;0.92] | 0.73 | 0.09 | [0.54;0.92] | 0.73 | 7.93  (5220) | < .001 | [-0.55;0.91] |
| Condition (Dynamic Touch) | -0.10 | 0.09 | [-0.28;0.08] | -0.10 | 0.09 | [-0.28;0.08] | -0.10 | 0.09 | [-0.28;0.09] | -0.10 | -1.10  (5220) | 0.27 | [-0.28;0.08] |
| Condition (Static Touch) | 0.05 | 0.09 | [-0.14;0.23] | 0.05 | 0.01 | [-0.13;0.24] | 0.05 | 0.09 | [-0.13;0.24] | 0.05 | 0.54  (5220) | 0.59 | [-0.13;0.23] |
| Baseline attractivenesss | -0.18 | 0.01 | [-0.21;-0.15] | -0.18 | 0.13 | [-0.21;-0.15] | -0.18 | 0.01 | [-0.21;-0.15] | -0.18 | -13.57  (5220) | < .001 | [-0.21;-0.15] |
| Sex (male) | 0.12 | 0.13 | [-0.14;0.38] | 0.12 | 0.04 | [-0.13;0.36] | 0.12 | 0.13 | [-0.13;0.37] | 0.12 | 0.93(47) | 0.36 | [-0.14;0.37] |
| PSI-Q | 0.02 | 0.04 | [-0.06;0.10] | 0.02 | 0.01 | [-0.06;0.10] | 0.02 | 0.04 | [-0.06;0.10] | 0.02 | 0.50(47) | 0.62 | [-0.06;0.10] |
| MMQ Ability | 0.01 | 0.01 | [-0.01;0.02] | 0.01 | 0.01 | [-0.01;0.02] | 0.01 | 0.01 | [-0.01;0.02] | 0.01 | 1.16(47) | 0.25 | [-0.01;0.02] |
| MMQ Satisfaction | 0.00 | 0.01 | [-0.02;0.03] | 0.00 | 0.01 | [-0.02;0.03] | 0.00 | 0.01 | [-0.02;0.03] | 0.00 | 0.28(47) | 0.78 | [-0.02;0.03] |
| CFMT+ | 0.00 | 0.01 | [-0.01;0.02] | 0.01 | 0.01 | [-0.01;0.02] | 0.00 | 0.01 | [-0.01;0.02] | 0.01 | 0.42(47) | 0.68 | [-0.01;0.02] |
| fCFMT+ | 0.00 | 0.01 | [-0.02;0.01] | 0.00 | 0.01 | [-0.02;0.01] | 0.00 | 0.01 | [-0.02;0.01] | 0.00 | -0.29(47) | 0.77 | [-0.02;0.01] |
| Age | 0.00 | 0.01 | [-0.02;0.02] | 0.00 | 0.01 | [-0.02;0.02] | 0.00 | 0.01 | [-0.02;0.02] | 0.00 | 0.00(47) | 0.99 | [-0.02;0.02] |
| Dynamic Touch*Baseline | 0.03 | 0.01 | [-0.01;0.06] | 0.03 | 0.02 | [-0.01;0.06] | 0.03 | 0.02 | [-0.01;0.06] | 0.03 | 1.48  (5220) | 0.14 | [-0.01;0.06] |
| Static Touch* Baseline | -0.01 | 0.02 | [-0.04;0.03] | -0.01 | 0.02 | [-0.04;0.03] | -0.01 | 0.02 | [-0.04;0.03] | -0.01 | -0.40  (5220) | 0.67 | [-0.04;0.03] |

Table 67. Model fits for stepwise approach. Note: improved model fit for model including moderation is trivial, since the baseline rating is part of the difference.

|  | df | AIC | BIC | Likelihood ratio |
| --- | --- | --- | --- | --- |
| Empty model + covariates | 10 | 17825.10 | 17890.80 | / |
| Empty model + covariates + condition | 12 | 17837.69 | 17916.53 | 8.59, p = .01 |
| Empty model + covariates + condition + interaction | 15 | 17471.01 | 17569.55 | 372.68,  p < .001 |
| Empty model + covariates + condition + interaction + random slope | 20 | 17406.65 | 17538.03 | 74.37,  p < .001 |

Table 68. Bayes factors for differences in trustworthiness ratings after touch moderated by baseline rating. Note: technically this improvement in model fit for model including moderation is trivial, since the baseline rating is part of the difference.

|  | Prior: small effect | Prior: medium effect | Prior: large effect |
| --- | --- | --- | --- |
| BF | < .01 | < .01 | < .01 |

## Moderation by Baseline Rating: Trustworthiness Ratings

### Touch versus no touch

Table 69. Full models of differences in trustworthiness ratings after touch moderated by baseline rating. Item-level regression to be able to include the baseline rating as moderator.

|  | Bayesian (Prior: small effect) | | | Bayesian (Prior: medium effect) | | | Bayesian (Prior: large effect) | | | Frequentist | | | |
| --- | --- | --- | --- | --- | --- | --- | --- | --- | --- | --- | --- | --- | --- |
|  | Beta | SE | CI | Beta | SE | CI | Beta | SE | CI | Beta | T(df) | p | CI |
| Intercept | 2.01 | 0.15 | [1.72;2.32] | 2.01 | 0.15 | [1.72;2.31] | 2.01 | 0.15 | [1.72;2.31] | 2.01 | -13.41  (5267) | < .001 | [1.72;2.31] |
| Condition (Touch) | 0.04 | 0.12 | [-0.20;0.27] | 0.04 | 0.12 | [-0.18;0.27] | 0.04 | 0.12 | [-0.18;0.27] | 0.04 | 0.36  (5267) | 0.72 | [-0.19;0.27] |
| Baseline trustworthiness | -0.42 | 0.02 | [-0.45;-0.39] | -0.42 | 0.02 | [-0.45;-0.39] | -0.42 | 0.02 | [-0.45;-0.38] | -0.42 | -24.09  (5267) | < .001 | [-0.45;0.39] |
| Sex (male) | 0.14 | 0.22 | [-0.30;-0.56] | 0.15 | 0.22 | [-0.30;-0.59] | 0.15 | 0.22 | [-0.30; 0.57] | 0.15 | 0.66  (5267) | 0.66 | [-0.28;0.58] |
| PSI-Q | -0.04 | 0.07 | [-0.18;0.11] | 0.03 | 0.07 | [-0.18;0.11] | -0.03 | 0.07 | [-0.17;0.10] | -0.03 | -0.50  (5267) | 0.62 | [-0.17;0.10] |
| MMQ Ability | 0.02 | 0.01 | [0.00;0.04] | 0.02 | 0.01 | [0.00;0.04] | 0.02 | 0.01 | [0.00;0.04] | 0.02 | 2.09  (5267) | 0.04 | [0.00;0.04] |
| MMQ Satisfaction | -0.02 | 0.02 | [-0.06;0.02] | -0.02 | 0.02 | [-0.06;0.03] | -0.02 | 0.02 | [-0.06;0.02] | -0.02 | -0.89  (5267) | 0.38 | [-0.06;0.02] |
| CFMT+ | 0.01 | 0.01 | [-0.01;0.04] | 0.01 | 0.01 | [-0.01;0.04] | 0.01 | 0.01 | [-0.01;0.04] | 0.01 | 1.13  (5267) | 0.26 | [-0.01;0.04] |
| fCFMT+ | -0.01 | 0.01 | [-0.03;0.02] | -0.01 | 0.01 | [-0.03;0.02] | -0.01 | 0.01 | [-0.03;0.02] | -0.01 | -0.59  (5267) | 0.56 | [-0.03;0.04] |
| Age | -0.02 | 0.01 | [-0.05;0.01] | -0.02 | 0.01 | [-0.05;0.01] | -0.02 | 0.01 | [-0.05;0.01] | -0.02 | -1.37  (5267) | 0.17 | [-0.05;0.01] |
| Touch*Baseline trustworthiness | 0.00 | 0.02 | [-0.04;0.04] | 0.00 | 0.02 | [-0.04;0.04] | 0.00 | 0.02 | [-0.04;0.04] | 0.00 | 0.17  (5267) | 0.86 | [-0.04;0.04] |

Table 70. Model fits for stepwise approach. Note: improved model fit for model including moderation is trivial, since the baseline rating is part of the difference.

|  | df | AIC | BIC | Likelihood ratio |
| --- | --- | --- | --- | --- |
| Empty model + covariates | 10 | 19971.53 | 20037.23 | / |
| Empty model + covariates + condition | 11 | 19977.72 | 20049.99 | 4.19, p = 0.4 |
| Empty model + covariates + condition + interaction | 13 | 18847.89 | 18933.29 | 1133.83,  p < .001 |
| Empty model + covariates + condition + interaction + random slope | 15 | 18721.58 | 18820.12 | 130.31,  p < .001 |

Table 71. Bayes factors for differences in trustworthiness ratings after touch moderated by baseline rating. Note: technically this improvement in model fit for model including moderation is trivial, since the baseline rating is part of the difference.

|  | Prior: small effect | Prior: medium effect | Prior: large effect |
| --- | --- | --- | --- |
| BF | < .01 | < .01 | < .01 |

### Static versus dynamic versus no touch

Table 72. Full models of differences in trustworthiness ratings after touch moderated by baseline rating. Item-level regression to be able to include the baseline rating as moderator.

|  | Bayesian (Prior: small effect) | | | Bayesian (Prior: medium effect) | | | Bayesian (Prior: large effect) | | | Frequentist | | | |
| --- | --- | --- | --- | --- | --- | --- | --- | --- | --- | --- | --- | --- | --- |
|  | Beta | SE | CI | Beta | SE | CI | Beta | SE | CI | Beta | T(df) | p | CI |
| Intercept | 2.01 | 0.15 | [1.71;2.29] | 2.01 | 0.15 | [1.70;2.29] | 2.01 | 0.15 | [1.72;2.30] | 2.01 | 13.42  (5220) | < .001 | [1.72;2.30] |
| Condition (Dynamic Touch) | -0.18 | 0.14 | [-0.44;0.09] | -0.18 | 0.13 | [-0.44;0.08] | -0.18 | 0.13 | [-0.44;0.09] | -0.18 | -1.30  (5220) | 0.19 | [-0.45;0.09] |
| Condition (Static Touch) | 0.24 | 0.13 | [-0.02;0.51] | 0.24 | 0.13 | [-0.02;0.50] | 0.24 | 0.13 | [-0.03;0.50] | 0.24 | 1.78  (5220) | 0.08 | [-0.02;0.50] |
| Baseline attractivenesss | -0.42 | 0.02 | [-0.45;-0.38] | -0.42 | 0.02 | [-0.45;-0.38] | -0.42 | 0.02 | [-0.45;-0.38] | -0.42 | -24.09  (5220) | < .001 | [-0.45;-0.38] |
| Sex (male) | 0.15 | 0.22 | [-0.27;0.58] | 0.15 | 0.21 | [-0.27;0.57] | 0.15 | 0.23 | [-0.30;0.59] | 0.15 | 0.67(47) | 0.51 | [-0.29;0.59] |
| PSI-Q | -0.03 | 0.07 | [-0.17;0.12] | -0.04 | 0.07 | [-0.18;0.11] | -0.03 | 0.07 | [-0.18;0.10] | -0.04 | 0.50(47) | 0.62 | [-0.17;0.10] |
| MMQ Ability | 0.02 | 0.01 | [0.00;0.04] | 0.02 | 0.02 | [0.00;0.04] | 0.02 | 0.01 | [0.00;0.04] | 0.02 | 2.10(47) | 0.04 | [0.00;0.04] |
| MMQ Satisfaction | -0.02 | 0.02 | [-0.06;0.02] | -0.02 | 0.01 | [-0.06;0.02] | -0.02 | 0.02 | [-0.06;0.02] | -0.02 | -0.88(47) | 0.38 | [-0.06;0.02] |
| CFMT+ | 0.01 | 0.01 | [-0.01;0.04] | 0.01 | 0.01 | [-0.01;0.04] | 0.01 | 0.01 | [-0.01;0.04] | 0.01 | 1.13(47) | 0.26 | [-0.01;0.04] |
| fCFMT+ | -0.01 | 0.01 | [-0.03;0.02] | -0.01 | 0.01 | [-0.03;0.02] | -0.01 | 0.01 | [-0.03;0.02] | 0.01 | -0.59(47) | 0.56 | [-0.03;0.02] |
| Age | -0.02 | 0.01 | [-0.05;0.01] | -0.02 | 0.01 | [-0.05;0.01] | -0.02 | 0.01 | [-0.05;0.01] | -0.02 | -1.37(47) | 0.18 | [-0.05;0.01] |
| Dynamic Touch*Baseline | 0.03 | 0.02 | [-0.01;0.08] | 0.03 | 0.02 | [-0.01;0.07] | 0.03 | 0.02 | [-0.02;0.07] | 0.03 | 1.30  (5220) | 0.19 | [-0.02;0.08] |
| Static Touch* Baseline | -0.02 | 0.02 | [-0.07;0.02] | -0.02 | 0.02 | [-0.06;0.02] | -0.02 | 0.02 | [-0.06;0.02] | -0.02 | -0.87  (5220) | 0.39 | [-0.06;0.02] |

Table 73. Model fits for stepwise approach. Note: improved model fit for model including moderation is trivial, since the baseline rating is part of the difference.

|  | df | AIC | BIC | Likelihood ratio |
| --- | --- | --- | --- | --- |
| Empty model + covariates | 10 | 19971.53 | 20037.23 | / |
| Empty model + covariates + condition | 12 | 19968.96 | 20047.80 | 6.57, p = .04 |
| Empty model + covariates + condition + interaction | 15 | 18848.60 | 18947.14 | 1126.36, p < .001 |
| Empty model + covariates + condition + interaction + random slope | 20 | 18623.81 | 18755.20 | 234.79, p < .001 |

Table 74. Bayes factors for differences in trustworthiness ratings after touch moderated by baseline rating. Note: technically this improvement in model fit for model including moderation is trivial, since the baseline rating is part of the difference.

|  | Prior: small effect | Prior: medium effect | Prior: large effect |
| --- | --- | --- | --- |
| BF | < 0.01 | < 0.01 | < 0.01 |
